# Exploiting the Brønsted Acidity of Phosphinecarboxamides for the Synthesis of New Phosphides and Phosphines

**DOI:** 10.1002/chem.201501174

**Published:** 2015-04-17

**Authors:** Andrew R Jupp, Gemma Trott, Éléonore Payen de la Garanderie, James D G Holl, Duncan Carmichael, Jose M Goicoechea

**Affiliations:** [a] Department of Chemistry, University of Oxford, Chemistry Research Laboratory12 Mansfield Road, Oxford, OX1 3TA (UK); [b] Laboratoire de Chimie Moléculaire, École PolytechniqueCNRS, 91128 Palaiseau cedex (France)

**Keywords:** anions, carboxamides, phosphides, phosphines, phosphorus

## Abstract

We demonstrate that the synthesis of new *N*-functionalized phosphinecarboxamides is possible by reaction of primary and secondary amines with PCO^−^ in the presence of a proton source. These reactions proceed with varying degrees of success, and although primary amines generally afford the corresponding phosphinecarboxamides in good yields, secondary amines react more sluggishly and often give rise to significant decomposition of the 2-phosphaethynolate precursor. Of the new *N*-derivatized phosphinecarboxamides available, PH_2_C(O)NHCy (Cy=cyclohexyl) can be obtained in sufficiently high yields to allow for the exploration of its Brønsted acidity. Thus, deprotonating PH_2_C(O)NHCy with one equivalent of potassium bis(trimethylsilyl)amide (KHMDS) gave the new phosphide [PHC(O)NHCy]^−^. In contrast, deprotonation with half of an equivalent gives rise to [P{C(O)NHCy}_2_]^−^ and PH_3_. These phosphides can be employed to give new phosphines by reactions with electrophiles, thus demonstrating their enormous potential as chemical building blocks.

Primary phosphines (or phosphanes), PH_2_R, particularly those containing small alkyl groups, are typically extremely air sensitive, pyrophoric, miasmic and toxic. This makes their use in chemical synthesis difficult, unpleasant and potentially very hazardous.[1] Consequently, they are underemployed as chemical reagents despite their numerous applications as starting materials for asymmetric ligands,[2] enantiomerically pure phosphorus-containing compounds,[3] biomedical agents,[4] macromolecules[5] and polymers.[6] Recently, the dehydrocoupling of primary phosphines has also been shown to afford new polyphosphanes with interesting structural characteristics.[7] On account of the enormous chemical versatility of these small molecules, more “user-friendly” primary phosphines with reduced air and moisture sensitivity are highly desirable. In the last few years, the number of reported air-stable primary phosphines has grown rapidly through two strategies: 1) the use of bulky substituents for the kinetic stabilization of these species; and/or 2) the use of substituents, which electronically stabilize the phosphine by facilitating lone-pair delocalization.[1,8,9] These species are attractive reagents on account of their reactive P=H bonds, which permit further functionalization at the phosphorus atom.

We recently reported the synthesis of phosphinecarboxamide (PH_2_C(O)NH_2_), a new member of the relatively small family of air-stable primary phosphines, by reaction of the 2-phosphaethynolate anion (PCO^−^) with ammonium salts.[10] Unlike other primary phosphines with similar properties, the relative stability of this molecule is not a result of the steric bulk of the carboxamide moiety, nor does it seem to arise due to a lack of lone-pair character at the phosphorus atom (the calculated phosphorus orbital character of the HOMO is 44.38 %). The PCO^−^ anion was first reported by Becker and co-workers and isolated as a lithium salt.[11] Due to the relative instability of this species, limited reactivity studies were reported in subsequent years.[12] But recently, several new syntheses of this remarkable anion have become available,[13] including a large-scale preparative method from inexpensive starting materials reported by Grützmacher and co-workers.[14] This has allowed the reactivity of this anion to be studied in much greater depth affording new heterocycles,[13a, [14,15] and low-valent phosphorus compounds.[16] Herein, we describe the synthesis of new *N*-functionalized phosphinecarboxamides and explore their reactivity for the synthesis of new phosphide anions and phosphines.

Small-scale reactions of PCO^−^ with primary amines (NH_2_R; R=Et, cyclohexyl (Cy), *t*Bu) in the presence of an acid gave new *N*-functionalized phosphinecarboxamides PH_2_C(O)NHR (R=Et (**1**), Cy (**2**) and *t*Bu (**3**)).[17] These species give rise to characteristic triplet resonances in their ^31^P NMR spectra, which collapse to singlets on proton decoupling. In contrast, the reactivity of PCO^−^ towards secondary amines (NHR_2_) does not proceed as readily, and we were only able to isolate H_2_PC(O)NEt_2_ (**4**). By analogy with the synthesis of urea,[18] the generation of phosphinecarboxamides using ammonium salts is believed to proceed through an initial protonation of the anion to afford the corresponding acid (HPCO), which we know to be unstable in solution. If the nucleophilic attack of the amine is slow, decomposition of the acid is observed, and the corresponding phosphinecarboxamide is not formed in suitable yields. A summary of selected spectroscopic data for **1**–**4** is available in Table 1. These new species are isoelectronic with the primary phosphaguanidine H_2_PC(NPh)NHPh previously reported by Issleib and co-workers.[19]

**Table 1:** Selected spectroscopic data for 1–4.

	^31^P [ppm]	^1^*J*_H=P_ [Hz]	^13^C [ppm]	^1^*J*_C=P_ [Hz]	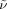 _CO_ [cm^−1^]
**1**	−136.7	205	171.3	6	1651
**2**	−135.8	206	170.7	6	1644
**3**	−134.8	206	171.3	7	1655
**4**	−125.4	218	172.9	8	1618

Of these new species, **2** is the most readily isolable and can be obtained as a compositionally pure solid in good yields (66 %). We were unable to obtain a crystalline sample suitable for single-crystal X-ray diffraction analysis; however, reaction of **2** with [Ru(*p*-cym)Cl_2_]_2_ (*p*-cym=*para*-cymene) gave the complex [Ru(*p*-cym){PH_2_C(O)NHCy}Cl_2_] (**5**), which allowed for the determination of structural metric data (Figure 1). Upon formation of **5**, there is a significant downfield shift of the ^31^P NMR resonance to −35.9 ppm, as well as an increase in the ^1^*J*_H=P_ coupling constant to 380 Hz (due to increased s orbital character of the P=H bonds), as was observed for other complexes of transition metals with phosphinecarboxamides.[20]

**Figure 1:**
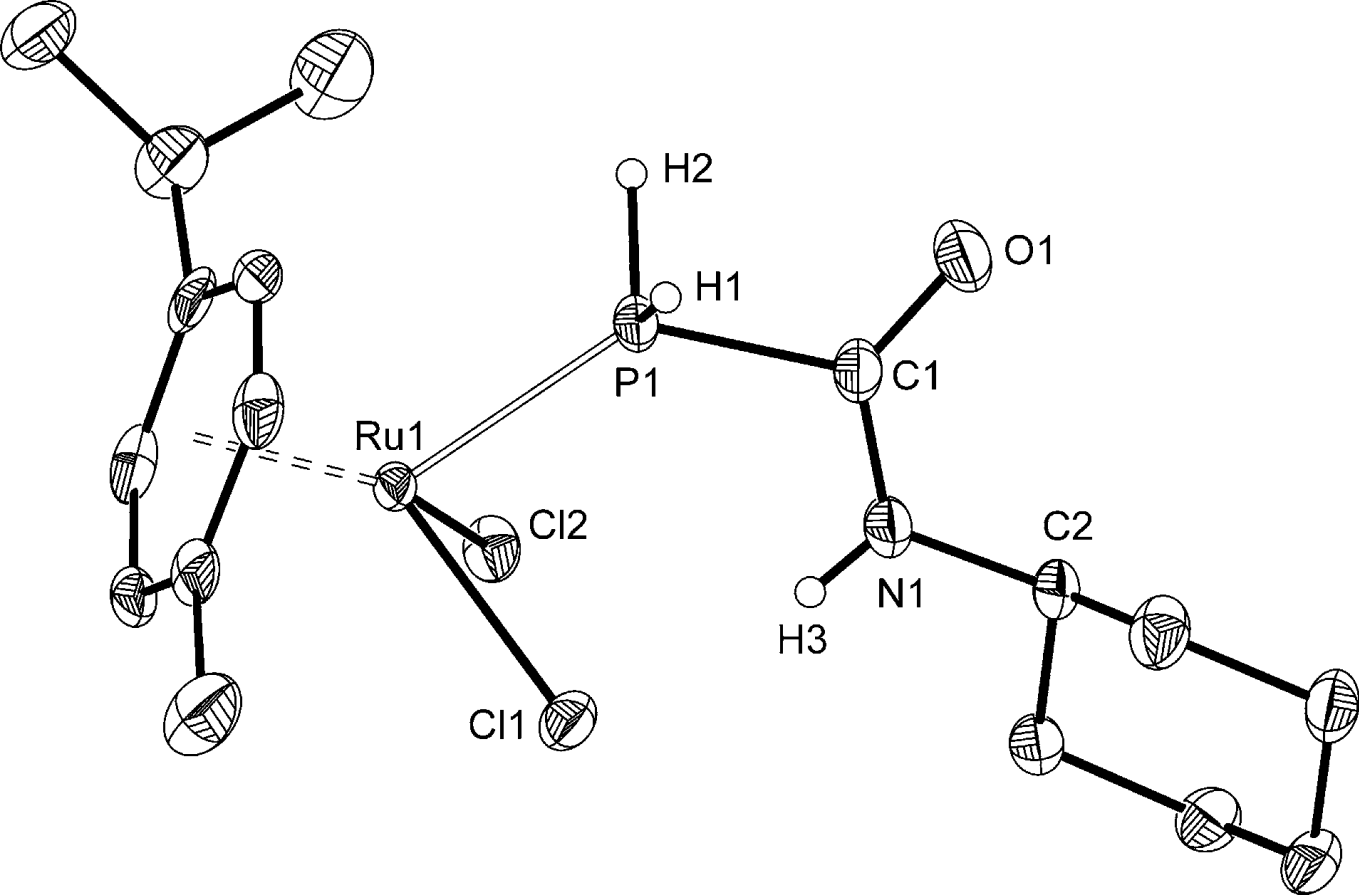
Molecular structure of 5. Anisotropic thermal displacement ellipsoids are pictured at the 50 % probability level. All hydrogen atoms (with the exception of those located in the Fourier difference map) have been removed for clarity. Selected interatomic distances [Å] and angles [°]: Ru1=P1: 2.318(1); Ru1=Cl1: 2.412(1); Ru1=Cl2: 2.413(1); P1=C1: 1.878(3); P1=H1: 1.28(4); P1=H2: 1.24(4); C1=O1: 1.227(4); C1=N1: 1.323(4); N1=C2: 1.461(4); N1=H3: 0.80(4); P1-C1-O1: 117.8(2); P1-C1-N1: 116.7(2); O1-C1-N1: 125.5(3).

The structural characterization of **5** permitted the determination of bond metric data for the phosphinecarboxamide moiety. The P=C, C=O and C=N bond lengths 1.878(3), 1.227(4) and 1.323(4) Å, respectively, are closely related to those recorded for the parent protic species (P=C: 1.865(1) Å; C=O: 1.230(2) Å; C=N: 1.329(2) Å).[10]

In an effort to establish the versatility of **1**–**4** as chemical building blocks, we were interested to explore their relative acidity. Reaction of **2** with one equivalent of potassium bis(trimethylsilylamide) (KHMDS) in the presence of 1,4,7,10,13,16-hexaoxacyclooctadecane ([18]crown-6) gives rise to clean deprotonation at the phosphorus atom affording [PHC(O)NHCy]^−^ (**6**) with no evidence of proton loss at the carboxamide moiety. This is appreciable in the appearance of a single doublet resonance in the ^31^P NMR spectrum (−97.3 ppm, ^1^*J*_H=P_=148 Hz), which collapses to a singlet on proton decoupling. Although it might appear counterintuitive that deprotonation should occur at the least polarized of the E=H bonds (E=N, P), the resulting phosphide is more stable than the analogous amide (by 29.2 kJ mol^−1^ according to calculations; see the Supporting Information for further details). The added stability is in a large part due to the possibility of delocalizing some of the negative charge over the carbonyl moiety, which is manifested in the bond metric data obtained for **6** (see below). It is worth noting that deprotonation of the parent protic species, PH_2_C(O)NH_2_, results in the rapid formation of PH_3_ and NCO^−^.

Compound **6** was structurally characterized in K[18]crown-6 [**6**], confirming deprotonation at the phosphorus atom (Figure 2). A comparison of structural metrics between the anion and the neutral parent complex showed a significant shortening of the P=C bond (1.791(3) Å; c.f. 1.878(3) in **5**, Δ*d*_P=C_=0.09 Å) indicative of a greater degree of multiple bond character. Consequently, this results in moderately longer C=O and C=N bonds for the phosphide species, which are 1.260(4) and 1.371(4) Å, respectively (C=O: 1.227(4) Å; C=N: 1.323(4) Å in **5**). An additional manifestation of the increased P=C multiple bond character in **6** is the fact that the PHC(O)NH core of the molecule is largely planar (deviation from planarity 0.0876 Å). These data were supported by quantum chemical calculations at the DFT level, which showed a good agreement with the bond metric data obtained for the optimized computed geometry of **6**.[21] Moreover, inspection of the Kohn–Sham frontier orbitals showed that the HOMO is predominantly P=C π bonding in character and antibonding for the carbonyl moiety. An analysis of Hirshfeld charges indicated an even distribution of negative charge over the phosphorus and oxygen atoms (−0.502 and −0.444, respectively).

**Figure 2:**
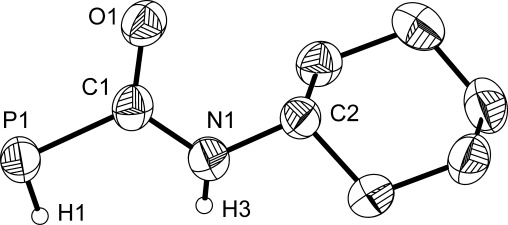
Structure of the anionic moiety characterized in K[18]crown-6 [6]. Anisotropic thermal displacement ellipsoids are pictured at the 50 % probability level. All hydrogen atoms (with the exception of those located in the Fourier difference map) have been removed for clarity. Selected interatomic distances [Å] and angles [°]: P1=C1: 1.791(3); P1=H1: 1.01(4); C1=O1: 1.260(4); C1=N1: 1.371(4); N1=C2: 1.454(4); N1=H3: 0.84(4); P1-C1-O1: 120.8(3); P1-C1-N1: 121.0(3); O1-C1-N1: 118.2(3).

Interestingly, deprotonation of **2** with half of an equivalent of KHMDS gave rise to a different phosphide anion, resulting from the nucleophilic attack of **6** at the remaining unreacted **2** (Scheme 1). The resulting species, [P{C(O)NHCy}_2_]^−^ (**7**), is an unprecedented phosphorus-containing anionic congener of biuret (NH{C(O)NH_2_}_2_), which is obtained through condensation of urea.[22] A related anionic species, [P{C(O)OMe}_2_]^−^, has previously been reported by Becker and co-workers, although it is worth noting that such species are generated using a different synthetic protocol.[23]

**Scheme 1:**
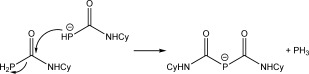
Synthesis of 7.

Compound **7** exhibits a broad singlet resonance in its ^31^P NMR spectrum at *δ*=−29.2 ppm and a doublet at 197.4 ppm (^1^*J*_C=P_=55 Hz) in the ^13^C spectrum. The ^1^H NMR spectrum revealed a broad singlet at 8.72 ppm (arising from the amide protons), as well as resonances arising from the cyclohexyl groups at *δ*=4.41 and 1.04–2.17 ppm, corresponding to the methine and methylene protons, respectively. The anion was structurally authenticated by single-crystal X-ray diffraction in K[18]crown-6 [**7**] (Figure 3). As would be expected, bond metric data indicate a lesser degree of delocalization of the phosphide π electrons into each adjacent carboxamide substituent when compared to **6**. Thus, the P=C bond lengths are 1.826(av) Å, which are in between the values recorded for the phosphinecarboxamide in **5** (1.878(3) Å) and **6** (1.791(3) Å). Accordingly, the C=O and C=N interatomic distances are not as short as those recorded for **6**. The structure of **7** revealed an intramolecular hydrogen-bonding interaction between one of the amide protons and a carbonyl oxygen atom with a distance of 1.88(4) Å. Computational studies on all of the possible isomers of **7** indicate that the conformation, as was determined in the crystal structure, is the most stable by 21.5 kJ mol^−1^. However, NMR spectroscopic data clearly indicate free rotation about the P=C bonds in solution, because both carboxamide moieties are magnetically equivalent.

**Figure 3:**
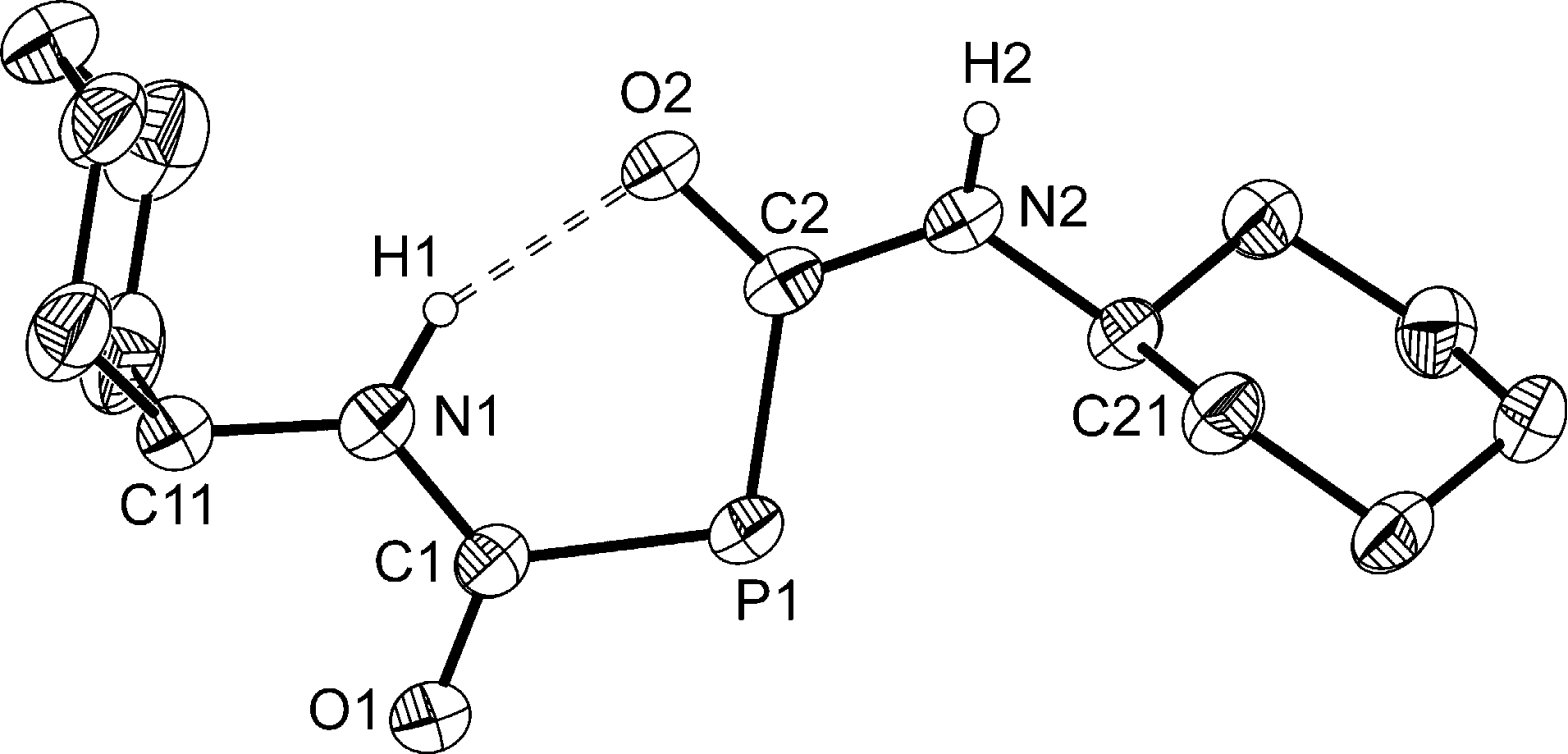
Structure of the anionic moiety characterized in K[18]crown-6 [7]. Anisotropic thermal displacement ellipsoids are pictured at the 50 % probability level. All hydrogen atoms (with the exception of those located in the Fourier difference map) have been removed for clarity. Selected interatomic distances [Å] and angles [°]: P1=C1: 1.825(2); C1=O1: 1.250(3); C1=N1: 1.357(3); N1=C11: 1.457(3); N1=H1: 0.93(4); P1=C2: 1.827(2); C2=O2: 1.266(3); C2=N2: 1.359(3); N2=C21: 1.459(3); N2=H2: 0.85(3); H1⋅⋅⋅O2: 1.88(4); P1-C1-O1: 116.8(2); P1-C1-N1: 121.3(2); O1-C1-N1: 121.8(2); P1-C2-O2: 126.2(2); P1-C2-N2: 116.0(2); O2-C2-N2: 117.8(2).

Protonation of K[18]crown-6 [**7**] using pyridinium trifluoromethanesulfonate readily gave the phosphorus-containing analogue of biuret HP{C(O)NHCy}_2_ (**8**). This species was identified by means of ^31^P NMR spectroscopy revealing a doublet resonance at *δ*=−74.9 ppm (^1^*J*_H=P_=235 Hz), which appears as a singlet in the proton-decoupled spectrum. The ^1^H NMR spectrum reveals a doublet at *δ*=4.95 ppm corresponding to the phosphine proton in addition to resonances arising from the amide substituents. The carbonyl ^13^C NMR resonance was recorded at 172.9 ppm and exhibited a ^1^*J*_C=P_ of 12 Hz.

Similarly, K[18]crown-6 [**6**] and K[18]crown-6 [**7**] can react with electrophiles, such as methyl iodide to afford the *P*-functionalized phosphines PH(CH_3_){C(O)NHCy} (**9**) and P(CH_3_){C(O)NHCy}_2_ (**10**). The former species exhibits a doublet of quartets in its ^31^P NMR spectrum at *δ*=−81.5 ppm consistent with methylation at the phosphorus atom. The methyl group was also observed in the ^1^H NMR spectrum as a doublet of doublets centred at *δ*=1.37 ppm along with resonances corresponding to the phosphine (4.05 ppm), amide (8.86 ppm) and cyclohexyl protons (4.16 and 0.80–2.10 ppm). Slow evaporation of a THF solution of **9** gave crystals suitable for single-crystal X-ray analysis (Figure 4). As expected, upon methylation of **6**, there was a notable elongation of the P=C(O) bond (from 1.791(3) to 1.863(3) Å) and a shortening of the C=O (1.239(3) Å) and C=N (1.323(3) Å) bonds (c.f. C=O: 1.260(4) Å; C=N: 1.371(4) Å in **6**). In other words, the structure of **9** closely resembles that of the parent *N*-functionalized phosphinecarboxamide **2**.

**Figure 4:**
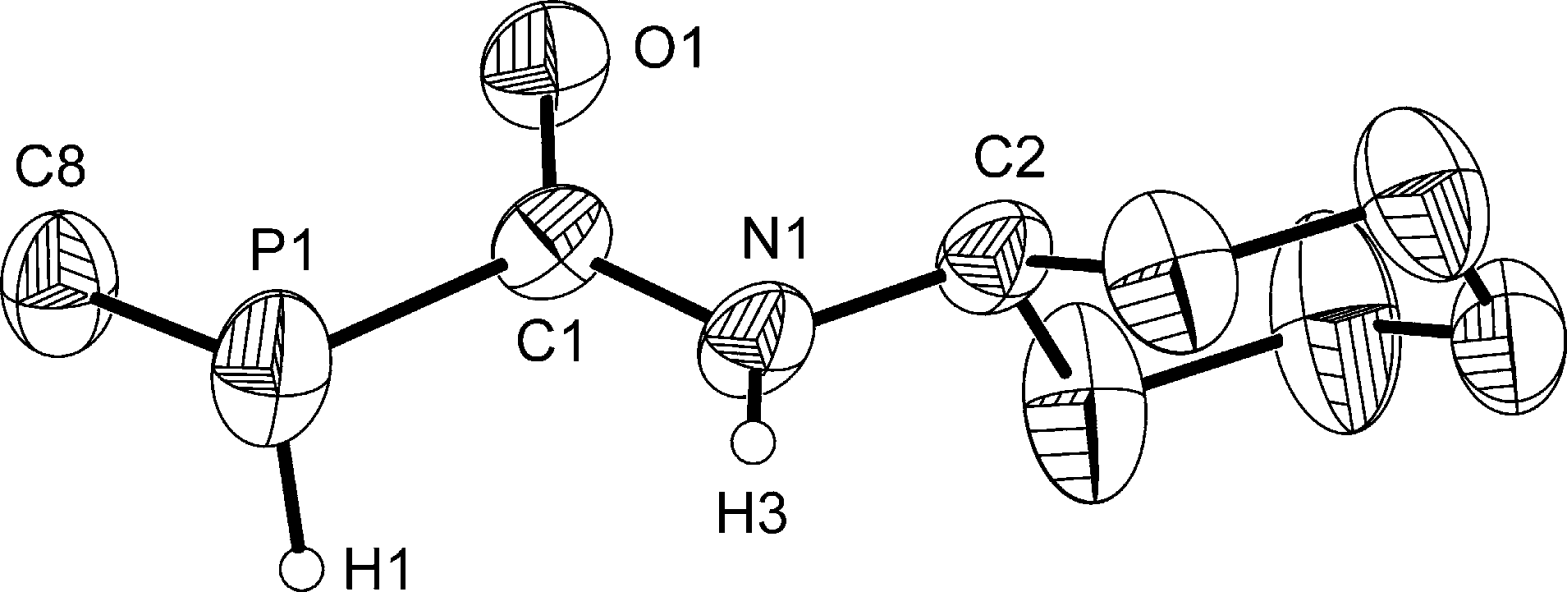
Structure of 9. Anisotropic thermal displacement ellipsoids are pictured at the 50 % probability level. All hydrogen atoms (with the exception of those located in the Fourier difference map) have been removed for clarity. Selected interatomic distances [Å] and angles [°]: P1=C1: 1.863(3); P1=C8: 1.824(3); P1=H1: 1.23(2); C1=O1: 1.239(3); C1=N1: 1.323(3); N1=C2: 1.462(4); N1=H2: 0.75(3); C1-P1-C8: 99.2(1); C1-P1-H1: 93.9(14); C8-P1-H1: 92.0(14); P1-C1-O1: 121.0(2); P1-C1-N1: 116.3(2); O1-C1-N1: 122.5(3).

Compound **10** exhibited a resonance in its ^31^P NMR spectrum at *δ*=−31.1 ppm. The ^1^H NMR spectrum is also as expected with resonances at *δ*=8.88 ppm for the amide protons, at 4.14 and 0.95–2.02 ppm for the cyclohexyl substituents, and a singlet resonance at *δ*=1.81 ppm arising from the phosphine methyl group. The ^13^C NMR spectrum of **10** is also entirely consistent with methylation at the phosphide exhibiting doublets for the carbonyl carbon atoms at *δ*=177.0 ppm (^1^*J*_C-P_=16 Hz), and for the methyl group at 7.2 ppm (^1^*J*_C=P_=12 Hz), as well as the requisite number of resonances for the cyclohexyl groups.

To summarise, we have shown that *N*-functionalized phosphinecarboxamides (a relatively new family of primary phosphines) may be used as precursors to secondary and tertiary phosphines by exploiting the relative acidity of the phosphine protons. These studies have yielded several species, which, in addition to their fundamental interest, show promise as supporting ligands for transition-metal complexes and as precursors to new molecules of potential industrial relevance.
