# Supplementary material for: Exploiting the Brønsted Acidity of Phosphinecarboxamides for the Synthesis of New Phosphides and Phosphines
Source: Chemistry. 2015 Apr 17;21(22):8015–8. doi: 10.1002/chem.201501174 (PMC4515089; doi:10.1002/chem.201501174)
Supplement: Supplementary file 1 [file chem0021-8015-sd1.pdf]

CHEMISTRY
A European Journal

Supporting Information

Exploiting the Brønsted Acidity of Phosphinecarboxamides for the Synthesis of New Phosphides and Phosphines

Andrew R. Jupp,[a] Gemma Trott,[a] Éléonore Payen de la Garanderie,[b] James D. G. Holl,[a] Duncan Carmichael,[b] and Jose M. Goicoechea*[a]

chem_201501174_sm_miscellaneous_information.pdf

Supporting Information

Contents

1. Experimental section

2. Single crystal X-ray diffraction data

3. Computation details

4. NMR spectra

5. MS spectra

6. IR spectra

7. References

1. Experimental section

1.1. General synthetic methods

All reactions were carried out in dry glassware under an inert atmosphere of argon or dinitrogen using standard Schlenk-line or glovebox techniques (MBraun UNIlab glovebox maintained at < 0.1 ppm H_2O and < 0.1 ppm O_2). Distillations were carried out in a fritted gas-tight H-cell under static vacuum. [K(18-crown-6)][PCO] and [Na(1,4-dioxane)$_x$][PCO] were prepared using literature reported methodologies.[1,2] Anhydrous dichloromethane (DCM; Fisher, HPLC grade) and hexane (Rathburn, 99+%) were purified through an MBraun SPS-800 solvent purification system before use. Tetrahydrofuran (THF; Rathburn, 99.9%) and pyridine (py; Alfa Aesar, 99+%) were distilled over a sodium metal/benzophenone mixture and CaH_2, respectively. [D_5]-Pyridine ([D_5]-py; Fluorochem, 99.5%), [D_8]-THF (Fluorochem, 99.5%) and [D_2]-dichloromethane (CD_2Cl_2; Eurisotop 99.9%) were dried over CaH_2 and vacuum distilled before use. Hexane, THF, diethyl ether, [D_5]-py, [D_8]-THF and CD_2Cl_2 were stored over activated 3 Å molecular sieves. All dry solvents were stored under argon in gas-tight ampoules. 18-crown-6 (1,4,7,10,13,16-hexaoxacyclooctadecane; Alfa Aesar, 99%), ethylamine hydrochloride ($EtNH_3Cl$; Alfa Aesar, 98%), were used after careful drying under vacuum and stored in the glovebox. Trifluoromethanesulfonic acid (triflic acid, HOTf; Alfa Aesar, 98+%) was used as received and stored under argon. Pyridinium trifluoromethanesulfonate (pyridinium triflate; Alfa Aesar, 97%), potassium bis(trimethylsilyl)amide (KHMDS; Sigma Aldrich, 95%), cyclohexylamine ($CyNH_2$; Alfa Aesar, 98+%), iodomethane (MeI; Sigma Aldrich, 99.5%) and $\{(p\text{-cymene})RuCl_2\}_2$ (Johnson Matthey) were all used as received and stored in the glovebox. Diethylamine (Et_2NH; Sigma Aldrich, 99+%) and *tert*-butylamine ($^tBuNH_2$; Sigma Aldrich, 98%) were dried over CaH_2 and vacuum distilled before use and stored in the glovebox.

1.2 Characterisation techniques

1.2.1. NMR spectroscopy

NMR samples were prepared inside an inert atmosphere glovebox in NMR tubes fitted with a gas-tight valve. ^{1}H NMR spectra were recorded at either 499.93 MHz or 400.13 MHz on a Bruker AVIII 500 or a Bruker AVIII 400 NMR spectrometer, respectively. $^{13}C\{^{1}H\}$ NMR spectra were recorded at either 125.80 MHz or 100.61 MHz on a Bruker AVII 500 fitted with a cryoprobe or a Bruker AVIII 500 NMR spectrometer, respectively. ^{31}P NMR spectra were recorded on 202.38 MHz or 161.98 MHz on a Bruker AVIII 500 or a Bruker AVIII 400 NMR spectrometer, respectively. ^{1}H and ^{13}C NMR spectra are reported relative to TMS and referenced to the most downfield residual solvent resonance where possible ([D$_8$]-THF: δ_H = 3.58 ppm, δ_C = 67.57 ppm; [D$_5$]-py: δ_H = 8.74 ppm, δ_C = 150.35 ppm). Where the most downfield residual THF resonance had been obscured, the spectra were referenced to the most upfield residual solvent resonance ([D$_8$]-THF: δ_H = 1.73 ppm). ^{31}P NMR spectra were externally referenced to an 85% solution of H_3PO_4 in H_2O (δ = 0 ppm). Data analysis was performed using Bruker TopSpin 3.1 software.

1.2.2. Single crystal X-ray diffraction

Single crystal X-ray diffraction data were collected using an Oxford Diffraction Supernova dual-source diffractometer equipped with a 135 mm Atlas CCD detector. Crystals were mounted on micromount loops after selection under Paratone-N oil and quench-cooled using an Oxford Cryosystems open flow N_2 cooling device.[3] Data were collected at 150 K using mirror monochromated Cu K_α radiation (λ = 1.5418 Å). and processed using the CrysAlisPro package, including unit cell parameter refinement and inter-frame scaling (which was carried out using SCALE3 ABSPACK within CrysAlisPro).[4] Structures were

subsequently solved using direct methods or using the charge flipping algorithm as implemented in the program SUPERFLIP,[5] and refined on F^2 using the SHELXL 13-4 package.[6]

1.2.3. Infrared spectroscopy

Spectra were recorded on a Thermo Scientific Nicolet iS5 FT-IR spectrometer in absorbance mode. Volatile compounds were dissolved in THF and the sample added to an air-tight solution phase IR cell in the glovebox before data collection. Solid samples were collected in a Nujol mull.

1.2.4. Mass spectrometry

Electrospray ionisation mass spectrometry (ESI-MS) data in positive and negative ion modes were obtained from THF or DMF solutions (10–20 μM) on a Waters LCT Time of Flight mass spectrometer with a Z-spray source (150 °C source temperature, 200 °C desolvation temperature, 25 V cone voltage and capillary voltages of 3.2 kV (positive ion mode) and 2.4 kV (negative ion mode)). Samples were injected using a 1 mL SGE syringe and syringe pump at a rate of 10 μL min^{-1}.

Electron ionisation mass spectrometry (EI-MS) and field ionisation mass spectrometry (FI-MS) were used for neutral compounds. EI and FI spectra were obtained on the neat sample using a Waters GCT Time of Flight Mass Spectrometer with a temperature programmed solids probe inlet. The authors would like to thank Colin Sparrow for help and advice with mass spectrometry, particularly the FI-MS.

1.3. Synthesis of compounds

1.3.1. *N*-ethylphosphinecarboxamide (**1**)

[K(18-crown-6)][PCO] (20 mg, 0.06 mmol) and $EtNH_3Cl$ (5 mg, 0.06 mmol) were dissolved in 0.5 mL [D_8]-THF in a gas-tight NMR tube. The crude reaction mixture was monitored by ^1H and ^{31}P NMR spectroscopy to ensure all of the amine starting material had been converted to **1**. The solution was distilled using a trap-to-trap distillation at 50 °C under reduced pressure to give **1** in solution. ^1H NMR (499.93 MHz, [D_8]-THF, 298 K): δ (ppm) 7.47 (br s, 1H; N*H*), 3.45 (d, 2H, $^1J_{H–P}$ = 205 Hz; PH_2), 3.19 (m, 2H; CH_2), 1.07 (t, 3H, $^3J_{H-H}$ = 7 Hz; CH_3). $^1H\{^{31}P\}$ NMR (499.93 MHz, [D_8]-THF, 298 K): δ (ppm) 3.45 (s, 2H; PH_2), other resonances unchanged from ^1H NMR spectrum. ^{31}P NMR (202.38 MHz, [D_8]-THF, 298 K): δ (ppm) –136.7 (t, $^1J_{H–P}$ = 205 Hz; *P*H_2). $^{31}P\{^1H\}$ NMR (202.38 MHz, [D_8]-THF, 298 K): δ (ppm) −136.7 (s; *P*H_2). $^{13}C\{^1H\}$ NMR (125.80 MHz, [D_8]-THF, 298 K): δ (ppm) 171.3 (d, $^1J_{C–P}$ = 6 Hz; H_2P*C*(O)), 35.4 (s; *C*H_2), 15.3 (s; *C*H_3). IR ([D_8]-THF, cm^{-1}): 1651 (CO), other wavenumbers unassignable due to overlap with [D_8]-THF.

1.3.2. *N*-cyclohexylphosphinecarboxamide (**2**)

A pyridine solution (100 mL) of [Na(1,4-dioxane)$_{2.9}$][PCO] (7.85 g, 23.3 mmol) was added to a stirring pyridine solution (100 mL) of H_2NCy (1.67 mL, 14.5 mmol) and pyridinium triflate (5 g, 21.8 mmol). Volatiles were removed *in vacuo* and **2** extracted in toluene (100 mL). Solvent was removed *in vacuo* to yield **2** as an off-white powder (1.3 g). A second toluene extraction (100 mL) was carried out again to yield a further 220 mg. Combined yield 1.52 g (66% with respect to the amine). ^1H NMR (499.93 MHz, [D_8]-THF, 298 K): δ (ppm) 7.33 (br, 1H; N*H*), 7.22–7.05 (toluene), 3.72 (m, 1H; NC*H*), 3.44 (d, 2H, $^1J_{H–P}$ = 206 Hz; PH_2), 2.30 (toluene), 1.89–1.08 (10H; Cy). $^1H\{^{31}P\}$ NMR (499.93 MHz, [D_8]-THF; 298 K): δ (ppm) 3.44 (s, 2H; PH_2), other resonances unchanged from ^1H NMR

spectrum. ^{31}P NMR (202.38 MHz, [D_8]-THF): δ (ppm) –135.8 (t, $^{1}J_{H–P}$ = 206 Hz; *P*H_2). $^{31}P\{^{1}H\}$ NMR (202.38 MHz, [D_8]-THF; 298 K): δ (ppm) –135.8 (s; *P*H_2). $^{13}C\{^{1}H\}$ NMR (125.80 MHz, [D_8]-THF; 298 K): δ (ppm) 170.7 (d, $^{1}J_{C–P}$ = 6 Hz; H_2P*C*(O)), 49.9 (s; Cy), 34.1 (s; Cy), 26.7 (s; Cy), 26.2 (s; Cy). Anal. Calc. $C_7H_{14}OPN$: C 52.82, H 8.87, N 8.80. Found: C 52.12, H 8.65, N 8.51. FI-MS: Calc. $PONC_7H_{14}$ *m/z* = 159.0813. Found *m/z* = 159.0818. Also observe M+H at 160.0930. IR (THF, cm^{-1}): 1644 (CO), other wavenumbers unassignable due to overlap with THF. IR (solid, Nujol mull, cm^{-1}) 1606 (CO).

1.3.3. *N-tert*-butylphosphinecarboxamide (**3**)

[K(18-crown-6)][PCO] (51 mg, 0.14 mmol), H_2N^tBu (6 μL, 0.06 mmol) were dissolved in 0.5 mL [D_8]-THF in an NMR tube. HOTf (10 μL, 0.11 mmol) was added in 2 μL portions and monitored by NMR spectroscopy until the reaction had gone to completion. The solution was distilled at room temperature under reduced pressure to give a solution of **3**. ^{1}H NMR (400.13 MHz, [D_8]-THF; 298 K): δ (ppm) 7.05 (br, 1H; N*H*), 3.44 (d, 2H, $^{1}J_{H–P}$ = 206 Hz; P*H*$_2$), 1.30 (s, 9H; C*H*$_3$). $^{1}H\{^{31}P\}$ NMR (499.93 MHz, [D_8]-THF; 298 K): δ (ppm) 3.44 (s, 2H; P*H*$_2$), other resonances unchanged from ^{1}H NMR spectrum. ^{31}P NMR (161.98 MHz, [D_8]-THF; 298 K): δ (ppm) –134.8 (t, $^{1}J_{H–P}$ = 206 Hz; *P*H_2). $^{31}P\{^{1}H\}$ NMR (161.98 MHz, [D_8]-THF; 298 K): δ (ppm) −134.8 (s; *P*H_2). $^{13}C\{^{1}H\}$ NMR (100.61 MHz, [D_8]-THF, 298 K): δ (ppm) 171.3 (d, $^{1}J_{C–P}$ = 7 Hz; H_2P*C*(O)), 53.3 (s; *C*Me_3), 29.2 (s; *C*H_3). IR ([D_8]-THF, cm^{-1}): 1655 (CO), other wavenumbers unassignable due to overlap with [D_8]-THF.

1.3.4. *N,N*-diethylphosphinecarboxamide (**4**)

[K(18-crown-6)][PCO] (36 mg, 0.10 mmol), Et_2NH (5 µL, 0.05 mmol) and pyridinium triflate (23 mg, 0.10 mmol) were dissolved in 0.5 mL [D_5]-py in an air-tight NMR tube and monitored by NMR spectroscopy until the reaction had gone to completion. The solution was distilled at room temperature under reduced pressure to give **4** in solution. ^1H NMR (499.93 MHz, [D_5]-py, 298 K): δ (ppm) 3.86 (d, 2H, $^1J_{H–P}$ = 218 Hz; PH_2), 3.34 (q, 2H, $^3J_{H–H}$ = 7 Hz; CH_2), 3.13 (qd, 2H, $^3J_{H–H}$ = 7 Hz, $^4J_{H–P}$ = 2 Hz; CH_2), 1.02 (t, 3H, $^3J_{H-H}$ = 7 Hz; CH_3), 1.00 (t, 3H, $^3J_{H–H}$ = 7 Hz, CH_3). $^1H\{^{31}P\}$ NMR (499.93 MHz, [D_5]-py, 298 K): δ (ppm) 3.86 (s, 2H; PH_2), 3.13 (q, 2H, $^3J_{H–H}$ = 7 Hz; CH_2), all other resonances unchanged with regard to the ^1H NMR spectrum. ^{31}P NMR (202.38 MHz, [D_5]-py, 298 K): δ (ppm) –125.4 (t, $^1J_{H–P}$ = 218 Hz; *P*H_2). $^{31}P\{^1H\}$ NMR (202.38 MHz, [D_5]-py, 298 K): δ (ppm) –125.4 (s; *P*H_2). $^{13}C\{^1H\}$ NMR (125.80 MHz, [D_5]-py, 298 K): δ (ppm) 172.9 (d, $^1J_{C-P}$ = 8 Hz; H_2P*C*(O)), 44.6 (d, $^3J_{C–P}$ = 10 Hz; CH_2), 40.2 (s; *C*H_2), 14.7 (s; *C*H_3), 13.7 (s; *C*H_3). IR ([D_8]-THF, cm^{-1}): 1618 (CO), other wavenumbers unassignable due to overlap with [D_8]-THF.

1.3.5. [(*p*-cymene)$RuCl_2$(H_2PC(O)NHCy)] (**5**)

{(*p*-cymene)$RuCl_2$}$_2$ (38 mg, 0.06 mmol) was added as a solid to a stirring dichloromethane solution (3 mL) of **2** (20 mg, 0.13 mmol) and stirred for 1 hour. The solvent was removed *in vacuo* to yield an orange solid. Orange blocks suitable for single crystal X-ray diffraction were grown by slow diffusion of hexane into a THF solution of **5**. ^1H NMR (400.13 MHz, CD_2Cl_2, 298 K): δ (ppm) 7.66 (br d, 1H, $^3J_{H–H}$ = 6 Hz; N*H*), 5.60 (d, 2H, $^3J_{H–H}$ = 6 Hz; H_{ar}), 5.45 (d, 2H, $^3J_{H–H}$ = 6 Hz; H_{ar}), 5.30 (d, 2H, $^1J_{H–P}$ = 376 Hz; PH_2), 3.77 (m, 1H; NC*H*), 2.79 (sept, 1H, $^3J_{H–H}$ = 7 Hz; C*H*(CH_3)$_2$), 2.17 (s, 3H; CH_3), 1.24 (d, 6H, $^3J_{H–H}$ = 7 Hz; CH(CH_3)$_2$), 1.14–1.85 (10H; Cy). ^{31}P NMR (202.38 MHz,

CD_2Cl_2, 298 K): δ (ppm) –36.9 (t, $^{1}J_{H–P}$ = 376 Hz). $^{31}P\{^{1}H\}$ NMR (202.38 MHz, CD_2Cl_2, 298 K): δ (ppm) –36.9 (s). $^{13}C\{^{1}H\}$ NMR (100.62 MHz, CD_2Cl_2, 298 K): δ (ppm) 163.9 (d, $^{1}J_{C–P}$ = 52 Hz; H_2PC(O)), 107.4 (d, $J_{C–P}$ = 2.0 Hz; $CH(CH_3)_2C_{ar}$), 102.8 (d, $J_{C–P}$ = 2.3 Hz; CH_3C_{ar}), 87.2 (d, $J_{C–P}$ = 4.7 Hz; HC_{ar}), 87.0 (d, $J_{C–P}$ = 3.6 Hz; HC_{ar}), 50.2 (d, $J_{C–P}$ = 4.1 Hz; N*C*H), 32.8 (s; Cy), 31.5 (s; *C*H(CH_3)$_2$), 25.9 (s; Cy), 24.9 (s; Cy), 22.5 (s; CH(CH_3)$_2$), 18.9 (s; *C*H_3). Anal. Calc. $C_{17}H_{28}Cl_2NOPRu$ (465.36): C 43.88, H 6.07, N 3.01. Found: C 43.69, H 6.00, N 3.08. EI-MS Parent peak not observed, only *p*-cymene and fragmentation thereof: *m/z* = 134.1020 $(C_{10}H_{14})^+$, 119.0684 $(C_9H_{11})^+$, 91.0476 $(C_7H_7)^+$.

1.3.6. [K(18-crown-6)][HPC(O)NHCy] ([K(18-crown-6)][**6**])

A toluene solution (30 mL) of **2** (200 mg, 1.26 mmol) was added to a stirring toluene solution (30 mL) of KHMDS (251 mg, 1.26 mmol), and the orange solution was left stirring overnight. 18-crown-6 (332 mg, 1.26 mmol) was subsequently added as a solid to the solution. The solvent and volatiles were removed *in vacuo* to afford an orange solid (393 mg, 68%). Crystals suitable for single crystal X-ray diffraction were grown by slow diffusion of hexane into a THF solution of the reaction product. ^{1}H NMR (499.94 MHz, [D_5]-py; 298 K): δ (ppm) 5.94 (br s, 1H; N*H*), 4.17 (m, 1H; NC*H*), 3.53 (s, 24H; 18-crown-6), 2.86 (d, 1H, $^{1}J_{H–P}$ = 148 Hz; P*H*) 1.02–2.20 (10H; Cy). ^{31}P NMR (202.38 MHz, [D_5]-py, 298 K): δ (ppm) –97.3 (d, $^{1}J_{H–P}$ = 148 Hz). $^{31}P\{^{1}H\}$ NMR (202.38 MHz, [D_5]-py, 298 K): δ (ppm) –97.3 (s). $^{13}C\{^{1}H\}$ NMR (125.72 MHz, [D_5]-py, 298 K): δ (ppm) 202.9 (d, $^{1}J_{C-P}$ = 59 Hz; P*C*(O)), 70.8 (s; 18-crown-6), 49.7 (s; N*C*H), 35.3 (s; Cy), 35.0 (s; Cy), 26.8 (s; Cy), 26.3 (s; Cy) 25.9 (s; Cy). ESI-MS (DMF, negative mode): No observable peak. ESI-MS (DMF, positive mode): *m/z* 764.97 $\{[K(18\text{-crown-}6)]_2[\mathbf{6}]\}^+$. Anal. Calc. $C_{19}H_{37}KNO_7P$ (461.56): C 49.44, H 8.08, N 3.04. Found: C 49.20, H 8.06, N 3.37. IR (solid, Nujol mull, cm^{-1}) 1538 (CO).

1.3.7. [K(18-crown-6)][P(C(O)NHCy)$_2$] ([K(18-crown-6)][**7**])

A THF solution (50 mL) of KHMDS (76 mg, 0.38 mmol) and 18-crown-6 (100 mg, 0.38 mmol) was slowly added to a stirring THF solution (10 mL) of **2** (120 mg, 0.76 mmol) which was kept at –78 ˚C. The reaction mixture was left to warm up to room temperature over two hours to yield a pale yellow solution. Volatiles were removed *in vacuo* to yield an off-white solid (102 mg, 46% yield). Crystals suitable for single crystal X-ray diffraction were grown by slow diffusion of hexane into a THF solution of [K(18-crown-6)][**7**]. ^{1}H NMR (500.30 MHz, [D$_5$]-py, 298 K): δ (ppm) 8.72 (br s, overlapping with solvent resonance; N*H*), 4.41 (m, 2H; NC*H*), 3.50 (s, 24H; 18-crown-6), 1.04–2.17 (m, 20H; Cy). ^{31}P NMR (161.96 MHz, [D$_5$]-py, 298 K): δ (ppm) –29.2 (br). ^{13}C{^{1}H} NMR (125.81 MHz, [D$_5$]-py, 298 K): δ (ppm) 197.4 (d, $^{1}J_{C-P}$ = 55 Hz; P(*C*O)$_2$), 70.8 (s; 18-crown-6), 48.0 (s; N*C*H), 34.9 (s; Cy), 26.8 (s; Cy), 25.9 (s; Cy). ESI-MS (DMF, negative mode): *m/z* 283.14 [**7**]$^{-}$. ESI-MS (DMF, positive mode): *m/z* 890.34 {[K(18-crown-6)]$_2$[**7**]}$^{+}$. Anal. Calc. $C_{26}H_{48}KN_2O_8P$ (586.73): C 53.22, H 8.25, N 4.77. Found: C 53.02, H 8.29, N 4.73. IR (solid, Nujol mull, cm^{-1}) 1560 and 1537 (CO).

1.3.8. *N,N'*-dicyclohexylphosphinedicarboxamide (**8**)

A THF solution (10 mL) of KHMDS (37.6 mg, 0.188 mmol) was added to a stirring THF solution (10 mL) of **2** (60 mg, 0.377 mmol) at –78 ˚C, and the pale yellow solution was left stirring for two days at room temperature. A THF solution (5 mL) of pyridinium triflate (43.2 mg, 0.188 mmol) was added to the stirring solution to immediately give a white precipitate. The colourless solution was filtered to a Schlenk tube and the volatiles removed *in vacuo* to yield a colourless oily substance. Alternatively, pyridinium triflate (7 mg, 0.03 mmol) was added as a solid to a [D$_5$]-pyridine solution (0.5 mL) of [K(18-crown-6)][**7**] (19 mg, 0.03 mmol) in a gas-tight NMR tube to give a pale yellow solution

containing **8** and [K(18-crown-6)][OTf], though separation of this by-product proved more difficult. ^{1}H NMR (499.93 MHz, [D_5]-py, 298 K): δ (ppm) 9.37 (br s, 2H; N*H*), 4.95 (d, 1H, $^{1}J_{H-P}$ = 235 Hz; P*H*), 4.12 (m, 2H; NC*H*), 0.95–2.05 (m, 20H; Cy). ^{1}H{^{31}P} NMR (499.93 MHz, [D_5]-py, 298 K): δ (ppm) 4.95 (s, 1H; P*H*), other resonances unchanged. ^{31}P NMR (161.98 MHz, [D_5]-py, 298 K): δ (ppm) –74.9 (d, $^{1}J_{H-P}$ = 235 Hz). ^{31}P{^{1}H} NMR (161.98 MHz, [D_5]-py, 298 K): δ (ppm) –74.9 (s). ^{13}C{^{1}H} NMR (125.72 MHz, [D_5]-py, 298 K): δ (ppm) 172.9 (d, $^{1}J_{C-P}$ = 12 Hz; P*C*(O)), 49.7 (s; Cy), 33.4 (s; Cy), 26.1 (s; Cy), 25.4 (s, Cy). FI-MS: Calc. $PO_2N_2C_{15}H_{27}$ *m/z* = 284.1654. Found *m/z* = 284.1637. Also observe CONCy at 125.0839 (derived from McLafferty rearrangement), M+H at 285.1724, and M+HCONCy at 410.2477. This is consistent with formation of H-bonded dimers prior to or during the ionisation process. **8** slowly decomposes under ambient conditions in the solid state so a satisfactory elemental analysis was not obtained. IR (solid, Nujol mull, cm^{-1}) 1639 and 1524 (CO).

1.3.9. *N*-cyclohexyl-1-methylphosphinecarboxamide (**9**)

A THF solution (10 mL) of **2** (60 mg, 0.377 mmol) was added to a stirring THF solution (10 mL) of KHMDS (75.1 mg, 0.377 mmol) at –78 ˚C, and the yellow solution was left stirring for three hours. Iodomethane (23.5 µL, 0.377 mmol) was added via a 25 µL microsyringe to the stirring solution to immediately give a white precipitate in a colourless solution. The solution was filtered to a Schlenk tube and the volatiles removed *in vacuo* to yield a white solid. Crystals suitable for single crystal X-ray diffraction were grown from slow evaporation of a THF solution of **9**. Alternatively, the product could be obtained by addition of iodomethane (3 µL, 0.05 mmol) to a [D_8]-THF solution (0.5 mL) of [K(18-crown-6)][**6**] (22.2 mg, 0.05 mmol) in a gas-tight NMR tube to give a colourless solution containing a white precipitate. The solution contained a clean sample of **9** and [K(18-

crown-6)][I], but complete separation of this by-product proved more difficult. ^{1}H NMR (499.94 MHz, [D$_5$]-py, 298 K): δ (ppm) 8.86 (br s, 1H; N*H*), 4.16 (m, 1H; NC*H*), 4.05 (dq, $^{1}J_{P\text{–}H}$ = 207 Hz, $^{3}J_{H\text{–}H}$ = 8 Hz, 1H; P*H*), 1.37 (dd, $^{3}J_{H\text{–}H}$ = 8 Hz, $^{2}J_{P\text{–}H}$ = 3 Hz, 3H; CH_3), 0.80–2.10 (m, 10H; Cy). ^{1}H{^{31}P} NMR (499.94 MHz, [D$_5$]-py, 298 K): δ (ppm) 4.05 (q, $^{3}J_{H\text{–}H}$ = 8 Hz, 1H; P*H*), 1.37 (d, $^{3}J_{H\text{–}H}$ = 8 Hz, 3H; CH_3), other resonances remain unchanged. ^{31}P NMR (161.98 MHz, [D$_5$]-py, 298 K): δ (ppm) –81.5 (dq, $^{1}J_{P\text{–}H}$ = 207 Hz, $^{2}J_{P\text{–}H}$ = 3 Hz). ^{31}P{^{1}H} NMR (161.98 MHz, [D$_5$]-py, 298 K): δ (ppm) –81.5 (s). ^{13}C{^{1}H} NMR (125.72 MHz, [D$_5$]-py, 298 K): δ (ppm) 176.9 (d, $^{1}J_{C\text{-}P}$ = 10 Hz; P*C*(O)), 49.8 (s; Cy), 34.0 (s; Cy), 33.9 (s; Cy), 26.3 (s; Cy), 25.9 (s; Cy, two resonances very close together), 1.8 (d, $^{1}J_{C\text{-}P}$ = 8 Hz; PCH_3). FI-MS: Calc. $PONC_8H_{16}$ *m/z* = 173.0970. Found *m/z* = 173.0971. IR (solid, Nujol mull, cm^{-1}) 1605 (CO).

1.3.10. *N*,*N*'-dicyclohexyl-1-methylphosphinedicarboxamide (**10**)

A THF solution (10 mL) of KHMDS (37.6 mg, 0.188 mmol) was added to a stirring THF solution (10 mL) of **2** (60 mg, 0.377 mmol) at –78 °C, and the pale yellow solution was left stirring for two days at room temperature. Iodomethane (11.8 µL, 0.188 mmol) was added via a 25 µL microsyringe to the stirring solution to immediately give a white precipitate (KI). The colourless solution was filtered to a Schlenk tube and the volatiles removed *in vacuo* to yield a colourless oily substance, from which a white solid could be obtained by trituration with pentane (20 mL). Yield 25 mg (45%). Alternatively, the product could be obtained by addition of iodomethane (2 µL, 0.03 mmol) to a [D$_5$]-pyridine solution (0.5 mL) of [K(18-crown-6)][**7**] (18 mg, 0.03 mmol) in a gas-tight NMR tube to give a pale yellow solution. The solution contained a clean sample of **10** and [K(18-crown-6)][I], but separation of this by-product proved more difficult. ^{1}H NMR (499.94 MHz, [D$_5$]-py, 298 K): δ (ppm) 8.88 (br s, 2H; N*H*), 4.14 (m, 2H; NC*H*), 1.81 (d,

$^{2}J_{P–H}$ = 2 Hz, 3H; CH_3), 0.95–2.02 (m, 20H; Cy). $^{1}H\{^{31}P\}$ NMR (499.94 MHz, [D_5]-py, 298 K): δ (ppm) 1.81 (s, 3H; CH_3), other resonances remain unchanged. ^{31}P NMR (161.98 MHz, [D_5]-py, 298 K): δ (ppm) –31.1 (s). $^{13}C\{^{1}H\}$ NMR (100.63 MHz, [D_5]-py, 298 K): δ (ppm) 177.0 (d, $^{1}J_{C-P}$ = 16 Hz; P*C*(O)), 49.7 (s; Cy), 33.5 (s; Cy), 26.2 (s; Cy), 25.6 (s, Cy), 7.2 (d, $^{1}J_{C-P}$ = 12 Hz; P*C*H_3). FI-MS: Calc. $PO_2N_2C_{15}H_{27}$ *m/z* = 298.1810. Found *m/z* = 298.1801. IR (solid, Nujol mull, cm^{-1}) 1633 and 1519 (CO).

2. Single crystal X-ray diffraction data

Table S1. Selected X-ray data collection and refinement parameters for **5**, [K(18-crown-6)][**6**], [K(18-crown-6)][**7**] and **9**.

	**5**	[K(18-crown-6)][**6**]	[K(18-crown-6)][**7**]	**9**
Formula	$C_{17}H_{28}Cl_2NOPRu$	$C_{19}H_{37}KNO_7P$	$C_{26}H_{48}KN_2O_8P$	$C_8H_{16}NOP$
Fw [g mol^{-1}]	465.34	461.56	586.73	173.19
crystal system	monoclinic	monoclinic	monoclinic	monoclinic
space group	$P2_1/n$	$P2_1$	$I2/a$	$P2_1/c$
a (Å)	12.6503(2)	8.7166(2)	26.594(3)	10.6789(8)
b (Å)	11.8975(1)	14.4487(4)	8.7621(8)	10.2465(8)
c (Å)	13.0798(2)	9.9967(3)	28.905(6)	9.6873(7)
β (°)	97.945(1)	101.815(3)	113.490(11)	108.621(8)
V (Å^3)	1949.70(5)	1232.35(6)	6177.3(16)	1004.51(14)
Z	4	2	8	4
radiation, λ (Å)	Cu Kα, 1.54178			
T (K)	150(2)			
ρ_{calc} (g cm^{-3})	1.585	1.244	1.262	1.145
μ (mm^{-1})	9.824	2.810	2.386	2.026
reflections collected	10687	10504	17344	4500
independent reflections	16124	4910	6357	1852
parameters	220	271	351	108
R(int)	0.0274	0.0325	0.0543	0.0338
R1/wR2,[a] I ≥ 2σI (%)	2.99/7.59	4.03/11.09	5.57/14.24	5.95/13.28
R1/wR2,[a] all data (%)	3.13/7.72	4.24/11.42	7.08/15.63	7.24/14.07
GOF	1.031	1.032	1.073	1.093

[a] $R1 = [\Sigma||F_o| - |F_c||]/\Sigma|F_o|$; $wR2 = \{[\Sigma w[(F_o)^2 - (F_c)^2]^2]/[\Sigma w(F_o^2)^2]\}^{1/2}$; $w = [\sigma^2(F_o)^2 + (AP)^2 + BP]^{-1}$, where $P = [(F_o)^2 + 2(F_c)^2]/3$ and the A and B values are 0.0425 and 2.69 for **5**, 0.0750 and 0.17 for [K(18-crown-6)][**6**], 0.0845 and 0.03 for [K(18-crown-6)][**7**] and 0.0387 and 1.07 for **8**.

3. Computational details

All geometry optimizations were performed using the Amsterdam Density Functional package (ADF2013.01).[7] An augmented all-electron TZ2P Slater-type basis set of triple-ζ quality, extended with two polarization functions, was used to describe all atoms (ATZ2P). Geometry optimizations were performed using the hybrid Becke three-parameter functional with Lee-Yang-Parr correlation (B3LYP).[8–10] Relativistic effects were incorporated using the Zeroth Order Relativistic Approximation (ZORA).[11] The presence of cations in the crystal lattice was modelled by surrounding the anion with a continuum dielectric using COSMO.[12] The chosen dielectric constant ε = 7.58 corresponds to that of THF, although structural parameters are not strongly dependent on this choice. All structures were optimized using the gradient algorithm of Versluis and Ziegler.[13]

Coordinates [Å] for the optimized geometry of 2

**Atom**	***x***	***y***	***z***
1. P	–1.700288	–0.768138	–0.414952
2. H	–1.644701	–1.364275	0.869378
3. H	–2.356800	0.405055	0.019502
4. C	0.010398	0.015978	–0.430006
5. O	0.187285	1.041033	–1.091110
6. N	0.989096	–0.640440	0.216445
7. H	0.732540	–1.442963	0.769007
8. C	2.393582	–0.224367	0.250651
9. H	2.460614	0.641957	-0.405354
10. C	2.803054	0.190958	1.670239
11. H	2.641687	–0.656037	2.345846
12. H	2.156763	1.000490	2.012831
13. C	4.274239	0.616678	1.721763
14. H	4.403700	1.529899	1.132581
15. H	4.548319	0.866932	2.748449

16. C	5.198569	–0.475258	1.172766
17. H	6.232028	–0.123050	1.167013
18. H	5.165680	–1.344183	1.837806
19. C	4.780209	–0.902289	–0.238181
20. H	5.413180	–1.718264	–0.591894
21. H	4.933166	–0.066601	–0.928418
22. C	3.308806	–1.330844	–0.288798
23. H	3.017781	–1.579916	–1.310593
24. H	3.168296	–2.234313	0.313762

Table S2. Interatomic bond distances (Å) and angles (°) for $PH_2C(O)NHCy$ (**2**) as crystallographically characterized in [(*p*-cymene)$RuCl_2(H_2PC(O)NHCy)$] (**5**), and the optimized computed geometry of **2**.

	**2**	**2_{DFT}**
P1−C1	1.878(3)	1.882
P1−H1	1.28(4)	1.417
P1−H2	1.24(4)	1.413
C1−O1	1.227(4)	1.233
C1−N1	1.323(4)	1.344
N1−C2	1.461(4)	1.465
N1−H3	0.80(4)	1.008
P1−C1−O1	117.8(2)	118.8
P1−C1−N1	116.7(2)	117.0
N1−C1−O1	125.5(3)	124.0

Coordinates [Å] for the optimized geometry of *trans*-6

Atom	*x*	*y*	*z*
1. P	1.588194	–0.807516	0.166976
2. H	0.997947	–2.073893	0.469543
3. C	0.031453	0.097678	–0.119456
4. O	0.041638	1.312873	–0.434438
5. N	–1.171349	–0.558693	–0.011445
6. H	–1.139839	–1.496768	0.353340
7. C	–2.467338	0.108615	0.009146
8. H	–2.366667	0.991959	–0.621989
9. C	–3.544562	–0.808253	–0.581897
10. H	–3.270438	–1.081832	–1.602784
11. H	–3.575350	–1.737734	–0.001585
12. C	–4.927961	–0.146687	–0.557262
13. H	–5.676717	–0.841310	–0.944839
14. H	–4.923929	0.715796	–1.231766
15. C	–5.311941	0.319657	0.851538
16. H	–6.276294	0.832717	0.827270
17. H	–5.436498	–0.554299	1.499672
18. C	–4.237156	1.236856	1.444337
19. H	–4.499322	1.520155	2.466374
20. H	–4.195151	2.164915	0.864740
21. C	–2.858805	0.566920	1.424890
22. H	–2.095120	1.246667	1.805733
23. H	–2.866345	–0.307474	2.084478

Coordinates [Å] for the optimized geometry of *cis*-6

Atom	*x*	*y*	*z*
1. P	1.473498	–1.001868	0.221851
2. H	2.428456	0.008779	–0.075113
3. C	0.018332	0.041007	–0.162772
4. O	0.040200	1.235635	–0.536324
5. N	–1.193875	–0.601620	–0.033760

6. H	–1.172378	–1.519431	0.383685
7. C	–2.483948	0.075554	–0.047348
8. H	–2.354533	0.955963	–0.677171
9. C	–3.559697	–0.823989	–0.666824
10. H	–3.258884	–1.110418	–1.676807
11. H	–3.630625	–1.748775	–0.082285
12. C	–4.928350	–0.130833	–0.691224
13. H	–5.680209	–0.809457	–1.100794
14. H	–4.881196	0.728181	–1.368661
15. C	–5.348958	0.350866	0.702165
16. H	–6.297415	0.890145	0.642012
17. H	–5.520443	–0.516839	1.347708
18. C	–4.272586	1.242081	1.330709
19. H	–4.562128	1.532019	2.343614
20. H	–4.189166	2.168680	0.752940
21. C	–2.909048	0.541461	1.355839
22. H	–2.143692	1.204697	1.762277
23. H	–2.957289	–0.332906	2.014421

Coordinates [Å] for the optimized geometry of amide-6

**Atom**	***x***	***y***	***z***
1. P	–1.619329	–0.829185	–0.169671
2. H	–1.893335	–0.687274	1.216011
3. H	–2.428620	0.278716	–0.533416
4. C	0.081001	0.037008	–0.158304
5. O	0.150353	1.279185	–0.397597
6. N	1.023889	–0.829167	0.100133
7. C	2.387943	–0.311580	0.193323
8. H	2.480083	0.606726	–0.398940
9. C	2.750506	0.043367	1.645675
10. H	2.621721	–0.853011	2.262351
11. H	2.046139	0.789636	2.018537
12. C	4.190205	0.555139	1.770303

13. H	4.280630	1.506193	1.234997
14. H	4.426588	0.764073	2.817113
15. C	5.195478	–0.446214	1.189657
16. H	6.207058	–0.033566	1.230274
17. H	5.199681	–1.349746	1.808992
18. C	4.835449	–0.827117	–0.251674
19. H	5.529259	–1.584452	–0.626211
20. H	4.958503	0.051352	–0.894619
21. C	3.391103	–1.331766	–0.360601
22. H	3.145255	–1.554332	–1.402306
23. H	3.283460	–2.269476	0.195490

Table S3. Interatomic bond distances (Å) and angles (°) for the crystallographically characterized and the optimized computed geometries of **6**.

	**6**	***trans*-6$_{\mathbf{DFT}}$**	***cis*-6$_{\mathbf{DFT}}$**	**amide-6$_{\mathbf{DFT}}$**
P1−C1	1.791(3)	1.823	1.831	1.908
P1−H1	1.01(4)	1.430	1.422	1.419
P1−H2	N/A	N/A	N/A	1.420
C1−O1	1.260(4)	1.255	1.252	1.267
C1−N1	1.371(4)	1.374	1.378	1.306
N1−C2	1.454(4)	1.458	1.457	1.462
N1−H3	0.84(4)	1.007	1.009	N/A
P1−C1−O1	120.8(3)	120.9	126.3	119.5
P1−C1−N1	121.0(3)	119.8	114.4	110.1
N1−C1−O1	118.2(3)	119.3	119.2	130.4
TOTAL BONDING ENERGY (KJ mol^{-1})		–14680.85	–14678.86	–14651.84

Coordinates [Å] for the optimized geometry of 7

**Atom**	***x***	***y***	***z***
1. P	–0.112662	–0.122320	0.347533
2. C	0.877108	–1.682428	0.370918
3. O	0.238504	–2.746925	0.556947
4. N	2.213184	–1.676571	0.189320
5. H	2.621695	–0.751096	0.066533
6. C	3.050728	–2.873907	0.212780
7. H	2.406439	–3.705666	-0.065120
8. C	3.622955	–3.151094	1.617691
9. H	4.079770	–4.146234	1.610160
10. H	2.804450	–3.183191	2.338200
11. C	4.673473	–2.115526	2.034201
12. H	4.196284	–1.136862	2.141058
13. H	5.078938	–2.371828	3.015754
14. C	5.801674	–2.019143	1.000200
15. H	6.519081	–1.249006	1.293102
16. H	6.350883	–2.966887	0.978017
17. C	5.250183	–1.723467	–0.399684
18. H	6.060424	–1.716891	–1.132239
19. H	4.817651	–0.718470	–0.414026
20. C	4.189263	–2.752325	–0.812825
21. H	3.774679	–2.505584	–1.792120
22. H	4.663324	–3.733905	–0.906177
23. C	1.134991	1.207710	0.098492
24. O	2.373852	1.041954	–0.065627
25. N	0.682719	2.497745	0.106121
26. H	1.399302	3.184836	–0.084327
27. C	–0.689517	2.985010	0.173951
28. H	–1.301414	2.162028	0.546782
29. C	–0.787956	4.150552	1.169141
30. H	–0.089222	4.935268	0.857471
31. H	–0.465035	3.811307	2.155552

32. C	–2.206166	4.729477	1.235861
33. H	–2.881975	3.979892	1.660348
34. H	–2.222330	5.583638	1.916876
35. C	–2.716330	5.141607	–0.148854
36. H	–3.743706	5.505868	–0.078169
37. H	–2.112633	5.975056	–0.523260
38. C	–2.633460	3.974613	–1.138635
39. H	–2.954956	4.294489	–2.132776
40. H	–3.326687	3.187165	–0.825261
41. C	–1.213660	3.400741	–1.210994
42. H	–1.181324	2.539590	–1.880765
43. H	–0.535777	4.154367	–1.626763

Table S4. Interatomic bond distances (Å) and angles (°) for the crystallographically characterized and the optimized computed geometries of **7**.

	**7**	**7**$_{\mathbf{DFT}}$
P1−C1	1.825(2)	1.848
C1−O1	1.250(3)	1.255
C1−N1	1.357(3)	1.348
N1−C11	1.457(3)	1.461
P1−C2	1.827(2)	1.841
C2−O2	1.266(3)	1.261
C2−N2	1.359(3)	1.367
N2−C21	1.459(3)	1.458
P1−C1−O1	116.83(16)	116.4
P1−C1−N1	121.33(17)	121.7
N1−C1−O1	121.8(2)	121.9
P1−C2−O2	126.20(17)	126.1
P1−C2−N2	115.99(15)	117.2
O2−C2−N2	117.79(18)	116.7

4. NMR spectra

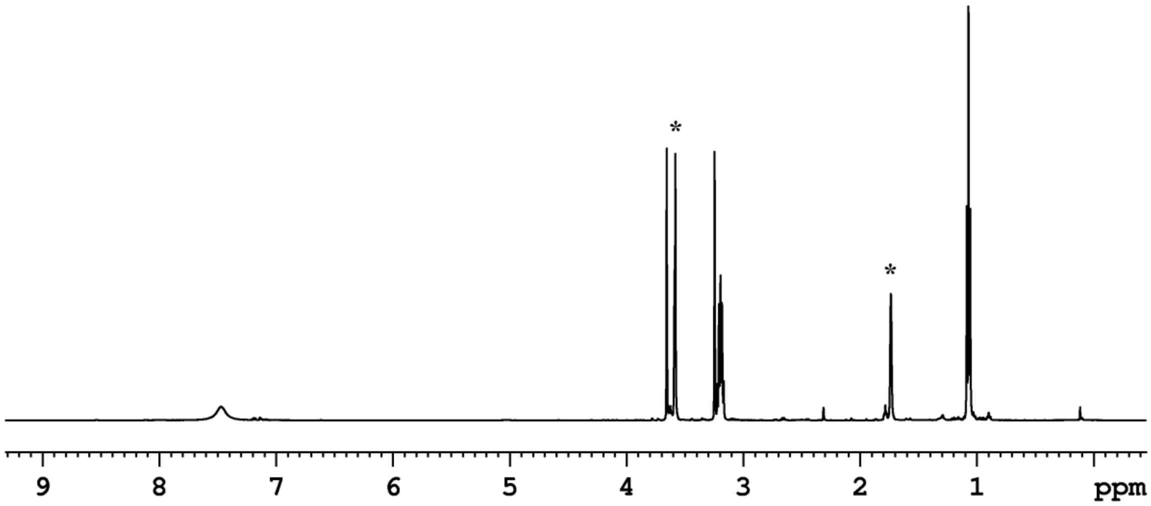

Figure S1. ^{1}H NMR spectrum of **1** in [D$_8$]-THF. Resonances marked with * are due to solvent.

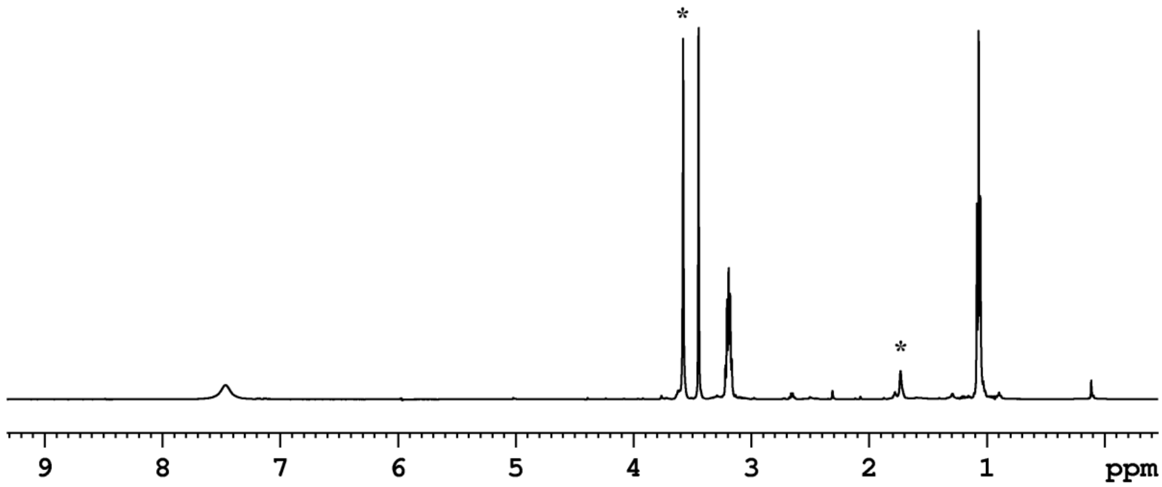

Figure S2. $^{1}H\{^{31}P\}$ NMR spectrum of **1** in [D$_8$]-THF. Resonances marked with * are due to solvent.

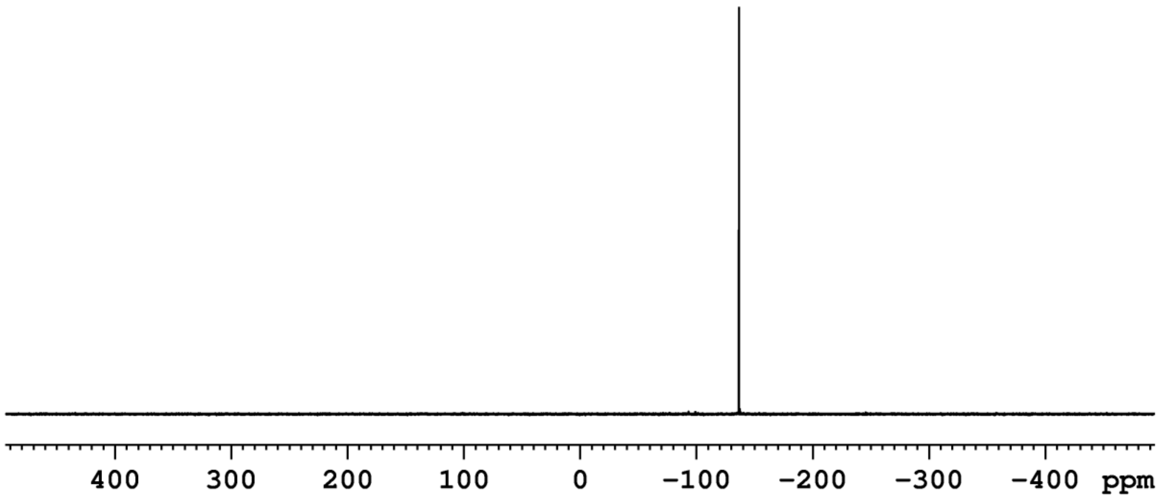

Figure S3. $^{31}P\{^1H\}$ NMR spectrum of **1** in [D$_8$]-THF.

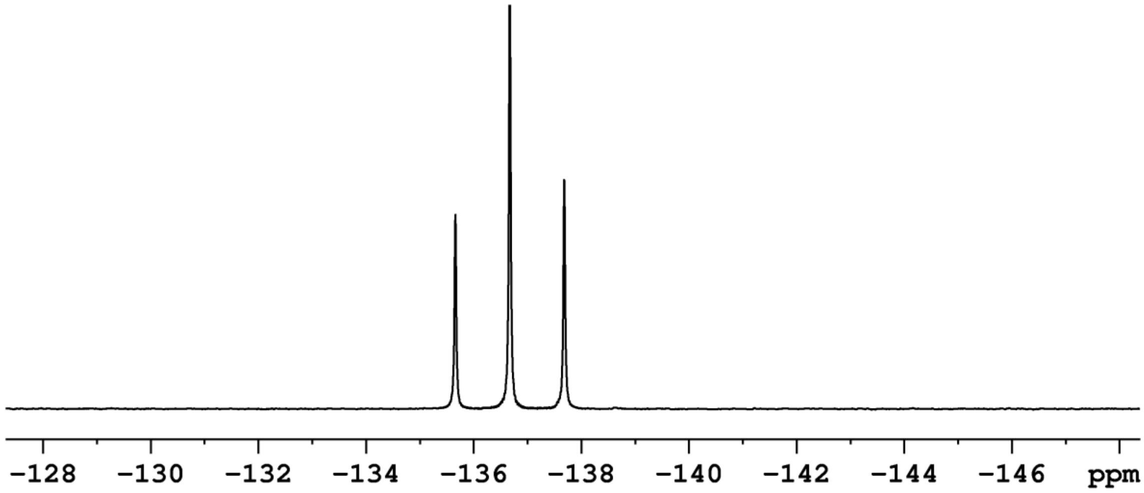

Figure S4. ^{31}P NMR spectrum of **1** in [D$_8$]-THF.

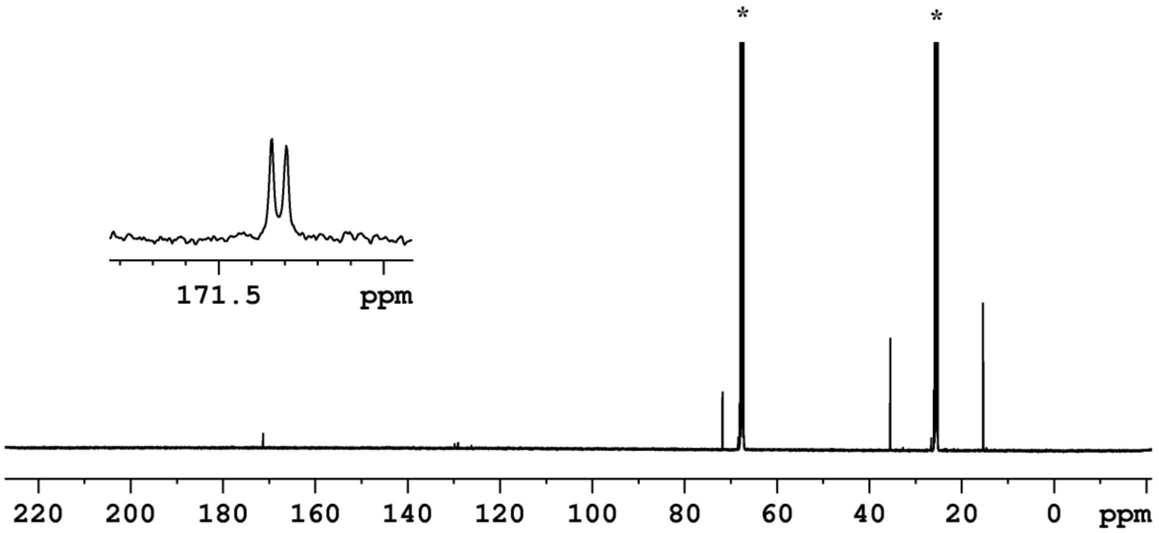

Figure S5. $^{13}C\{^{1}H\}$ NMR spectrum of **1** in [D_8]-THF. Resonances marked with * are due to solvent. Inset shows zoomed region of the carbonyl.

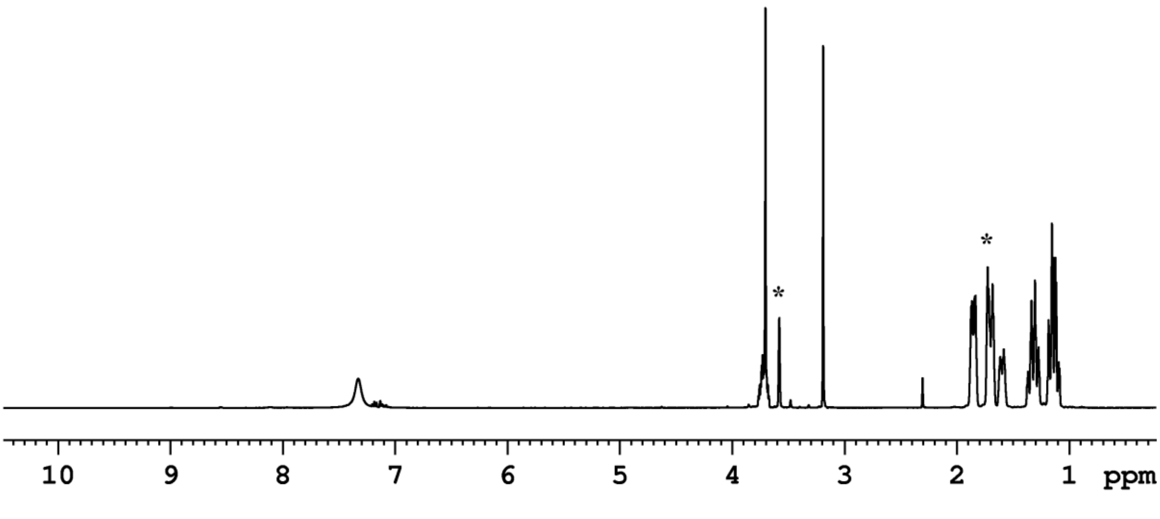

Figure S6. ^{1}H NMR spectrum of **2** in [D_8]-THF. Resonances marked with * are due to solvent.

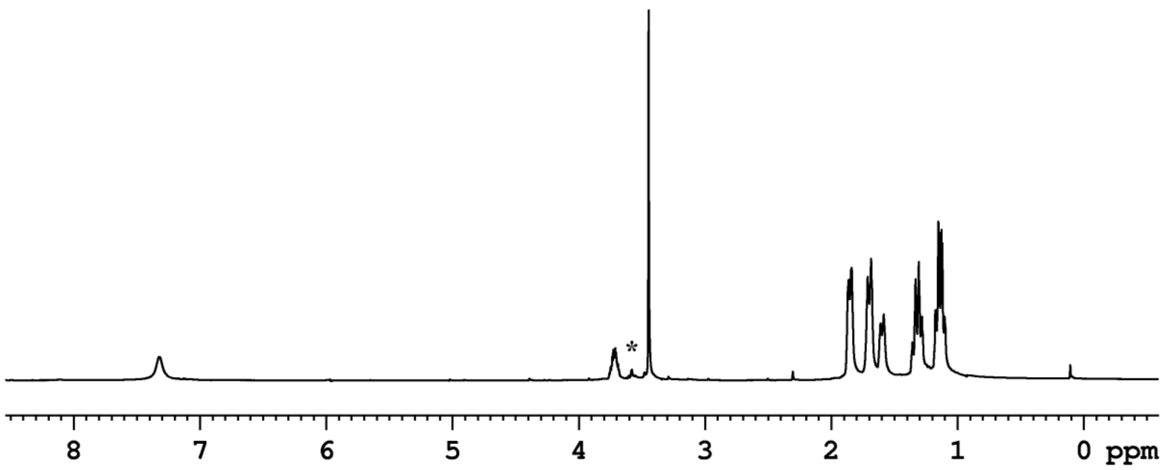

Figure S7. $^{1}H\{^{31}P\}$ NMR spectrum of **2** in [D_8]-THF. Resonance marked with * is due to solvent, high field solvent resonance obscured by cyclohexyl region.

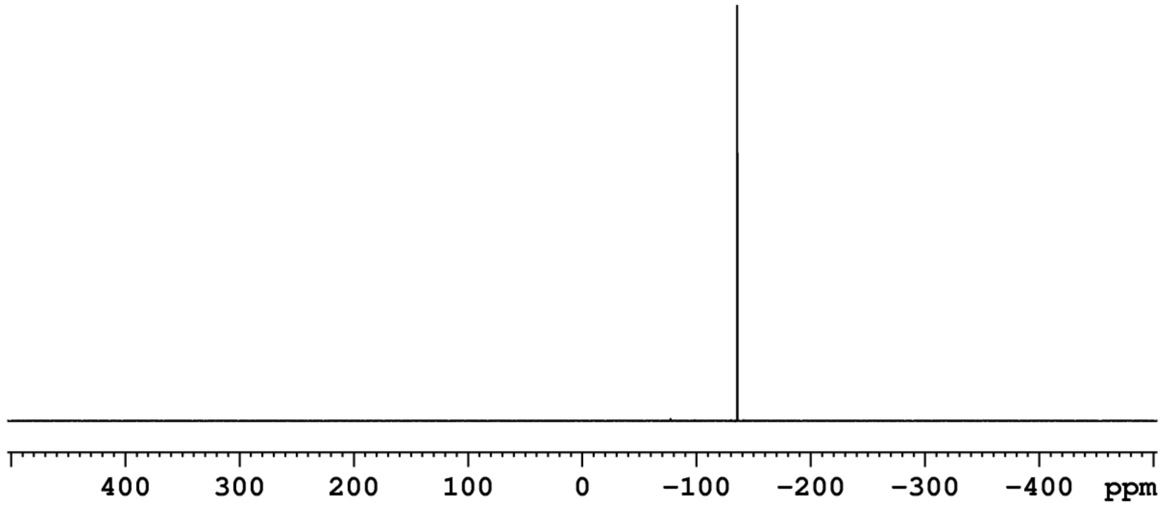

Figure S8. $^{31}P\{^{1}H\}$ NMR spectrum of **2** in [D_8]-THF.

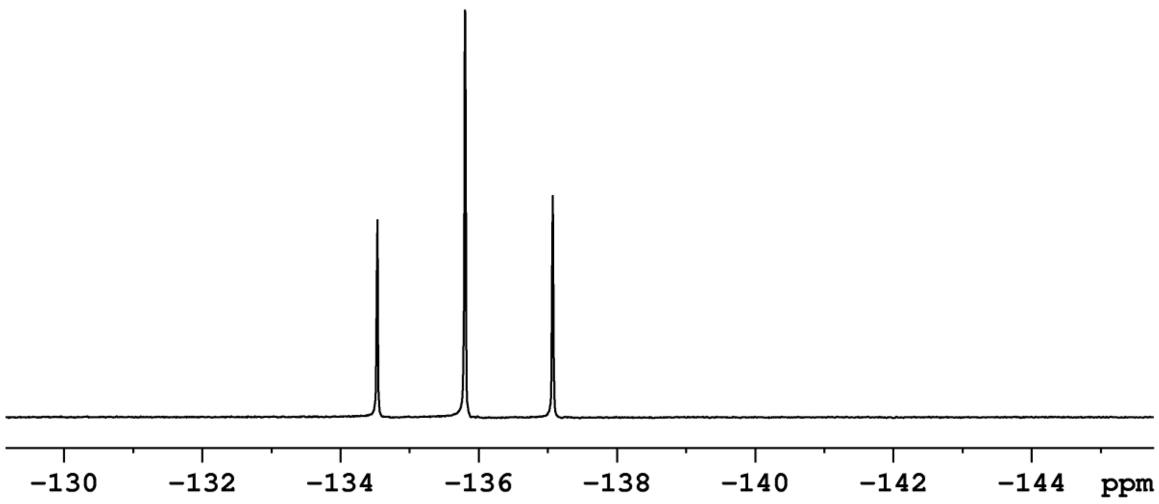

Figure S9. ^{31}P NMR spectrum of **2** in [D_8]-THF.

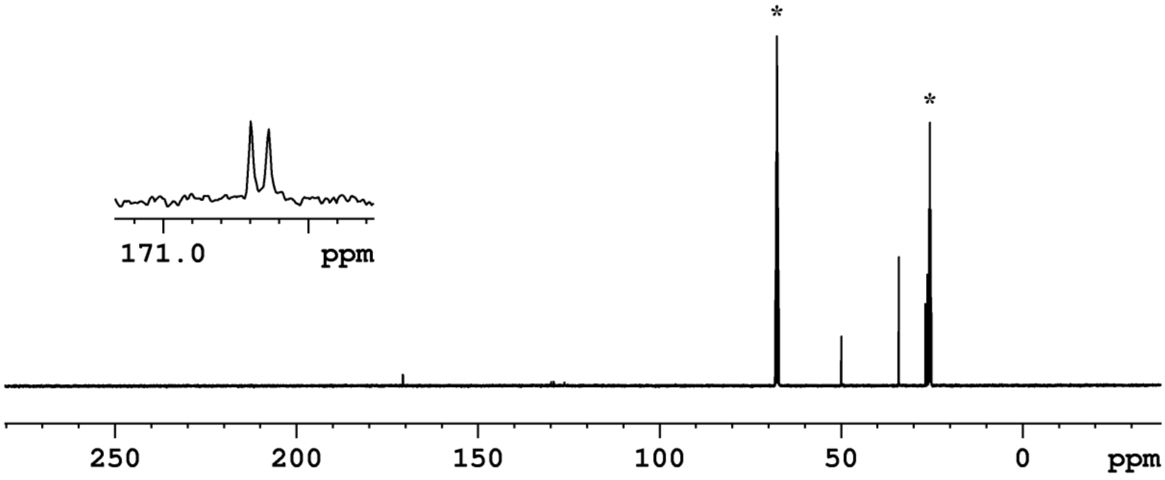

Figure S10. $^{13}C\{^1H\}$ NMR spectrum of **2** in [D_8]-THF. Resonances marked with * are due to solvent. Inset shows zoomed region of the carbonyl.

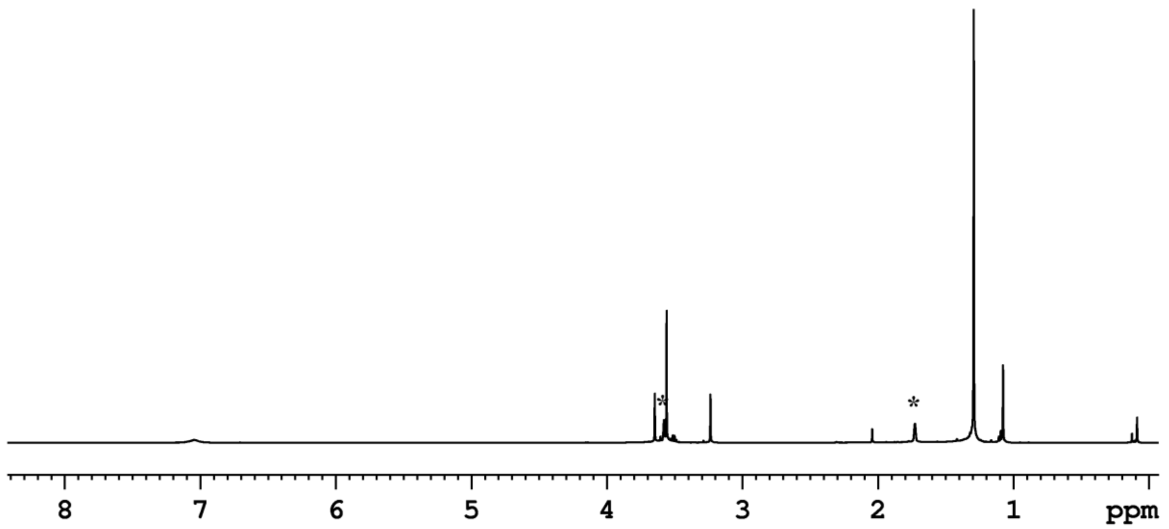

Figure S11. ^{1}H NMR spectrum of **3** in [D_8]-THF. Resonances marked with * are due to solvent.

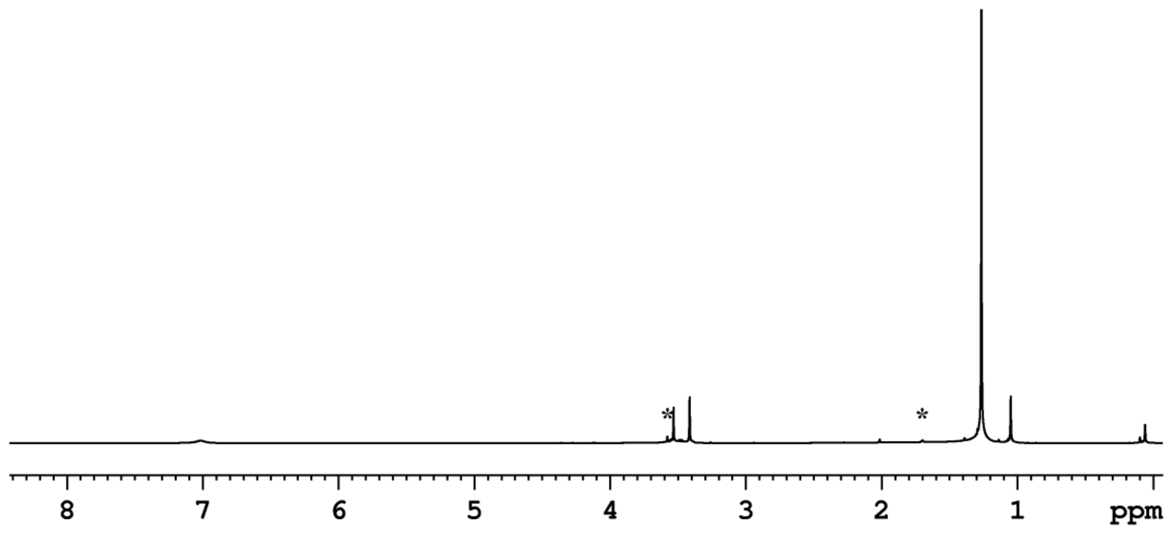

Figure S12. $^{1}H\{^{31}P\}$ NMR spectrum of **3** in [D_8]-THF. Resonances marked with * are due to solvent.

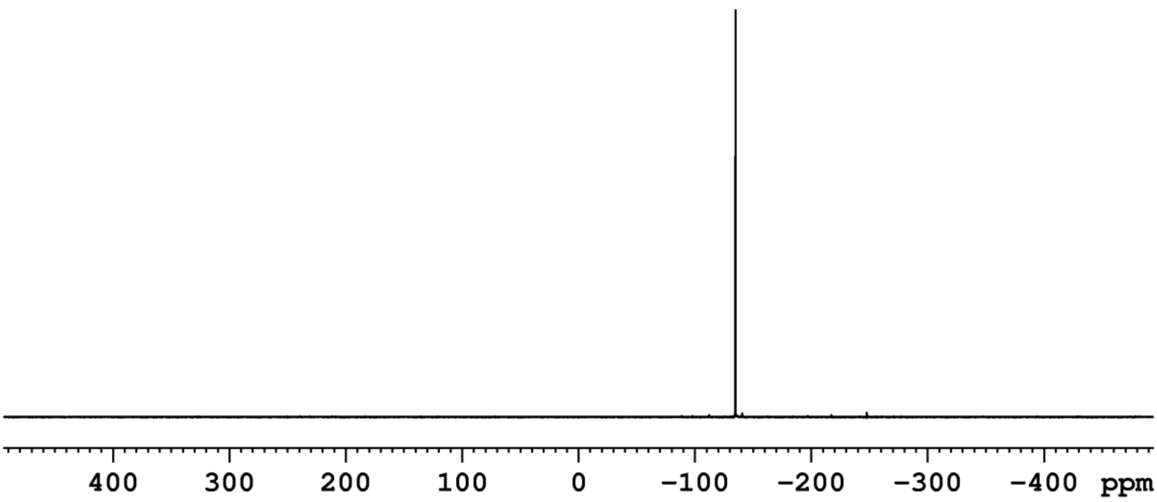

Figure S13. $^{31}P\{^{1}H\}$ NMR spectrum of **3** in [D_8]-THF.

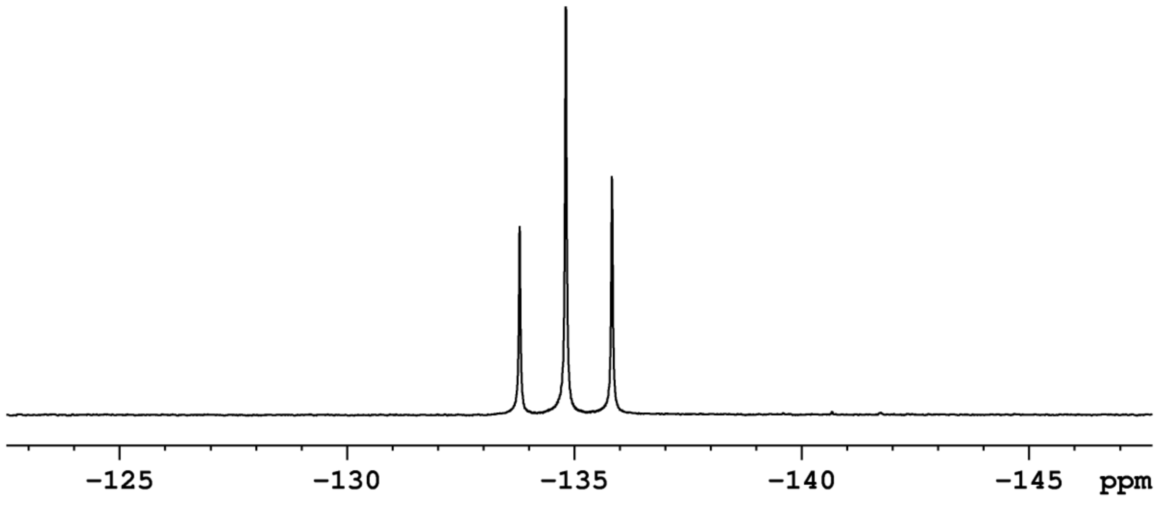

Figure S14. ^{31}P NMR spectrum of **3** in [D_8]-THF.

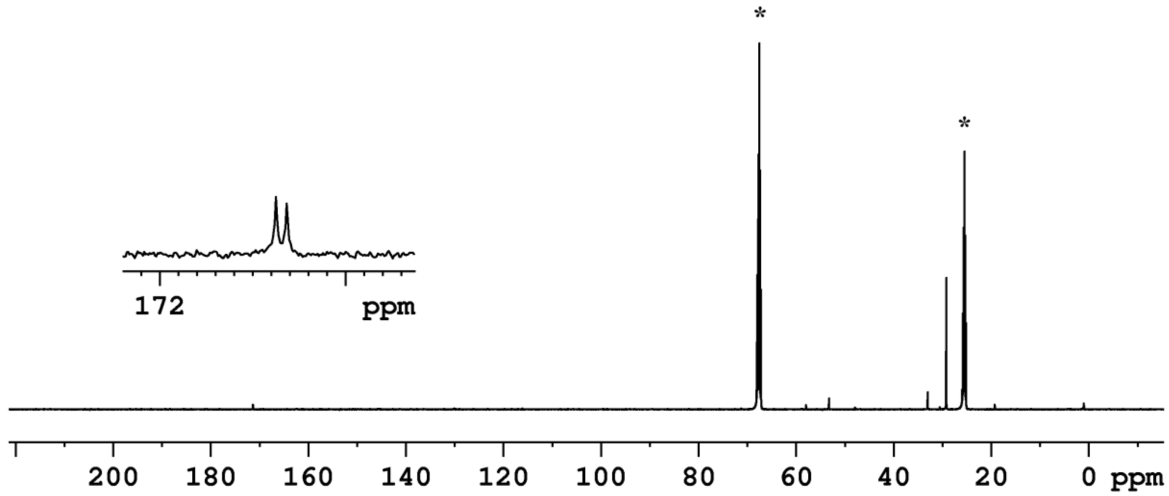

Figure S15. $^{13}C\{^{1}H\}$ NMR spectrum of **3** in [D$_8$]-THF. Resonances marked with * are due to solvent. Inset shows zoomed region of the carbonyl.

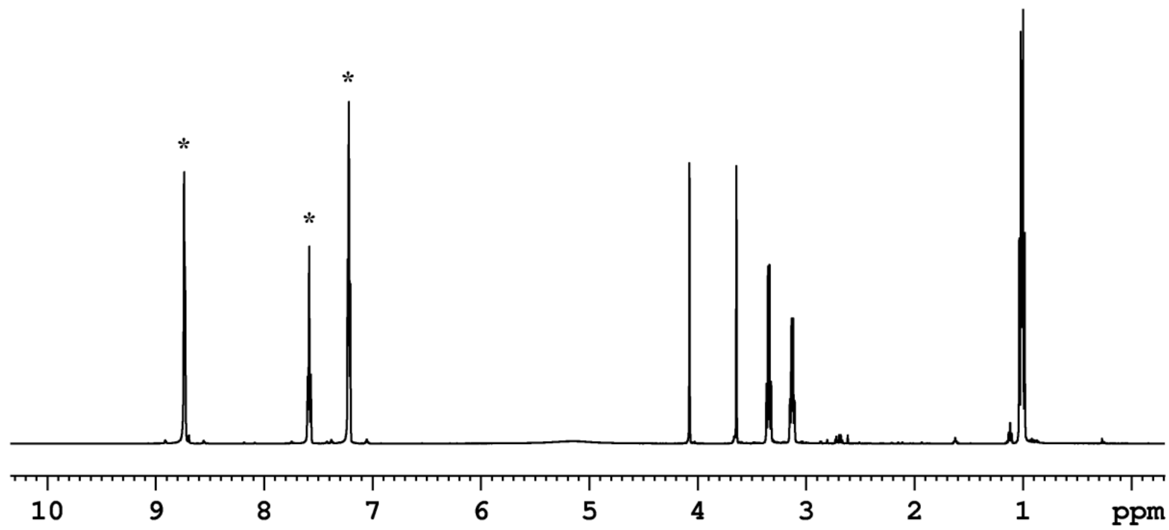

Figure S16. ^{1}H NMR spectrum of **4** in [D$_5$]-pyridine. Resonances marked with * are due to solvent.

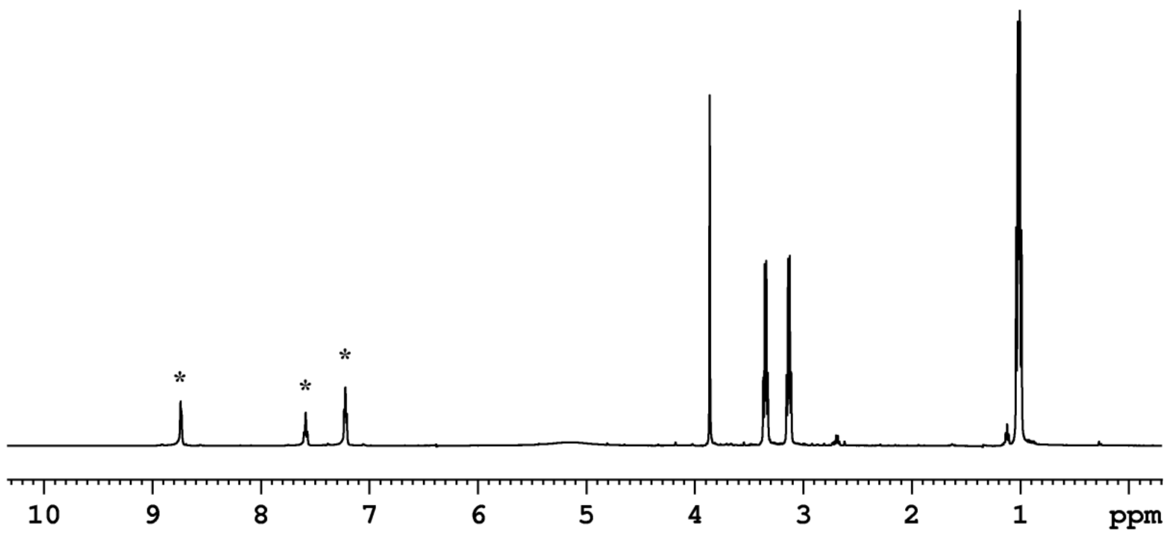

Figure S17. $^{1}H\{^{31}P\}$ NMR spectrum of **4** in [D_5]-pyridine. Resonances marked with * are due to solvent.

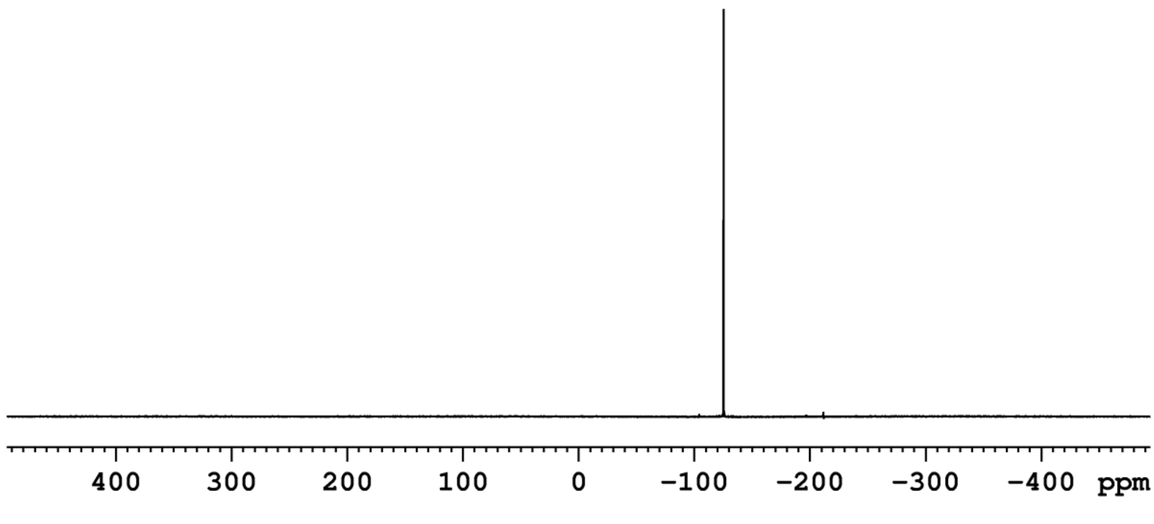

Figure S18. $^{31}P\{^{1}H\}$ NMR spectrum of **4** in [D_5]-pyridine.

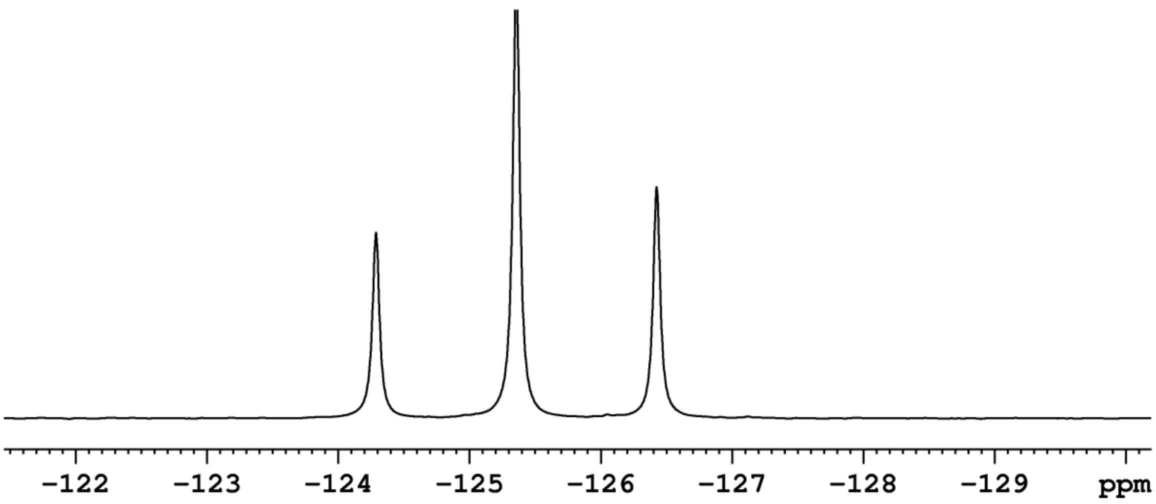

Figure S19. ^{31}P NMR spectrum of **4** in [D$_5$]-pyridine.

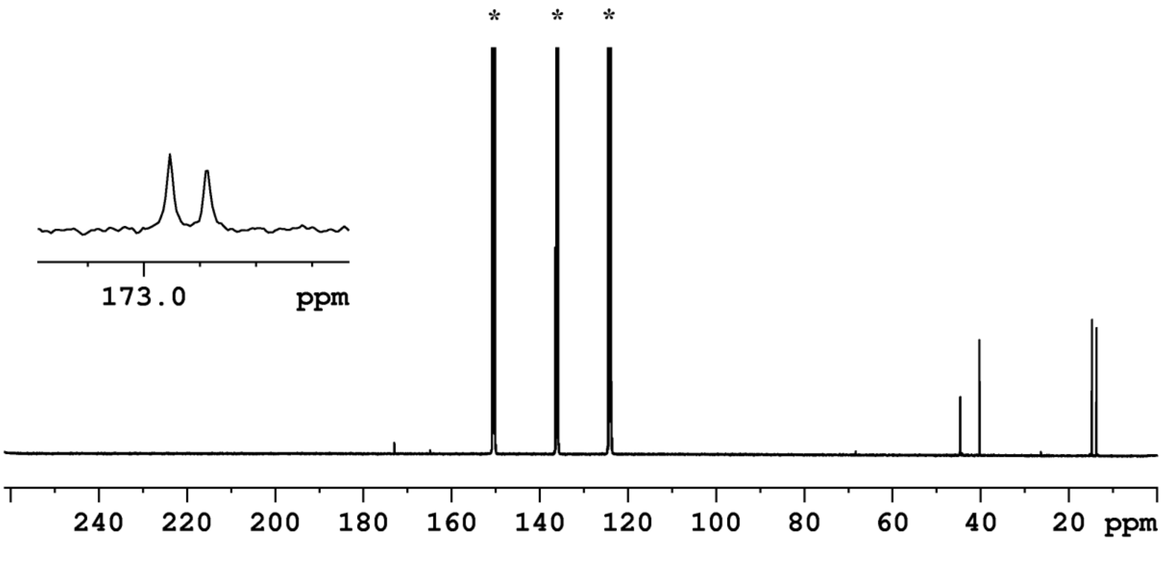

Figure S20. $^{13}C\{^{1}H\}$ NMR spectrum of **4** in [D$_5$]-pyridine. Resonances marked with * are due to solvent. Inset shows zoomed region of the carbonyl.

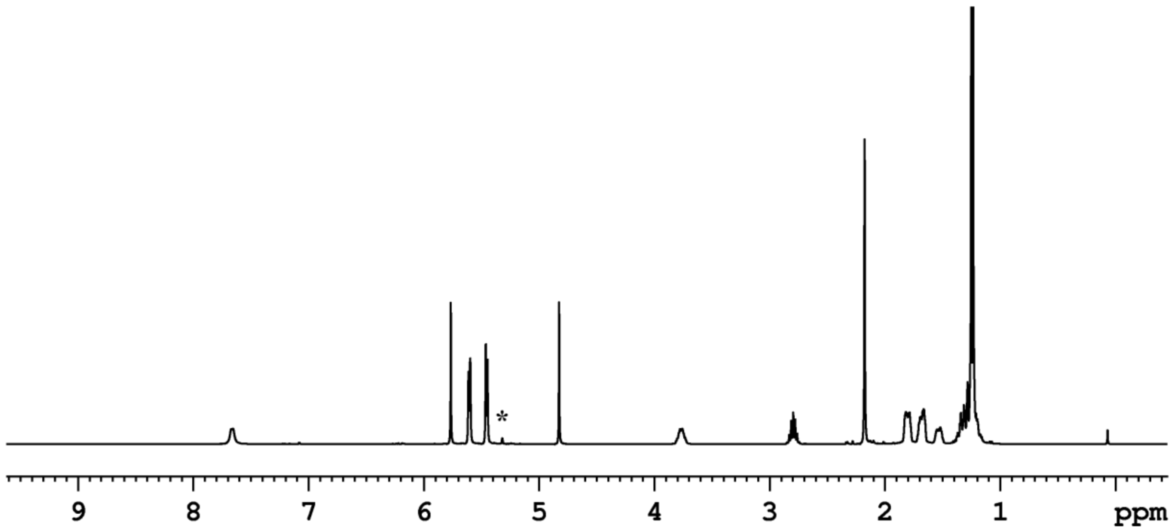

Figure S21. ^{1}H NMR spectrum of **5** in CD_2Cl_2. Resonance marked with * is due to solvent.

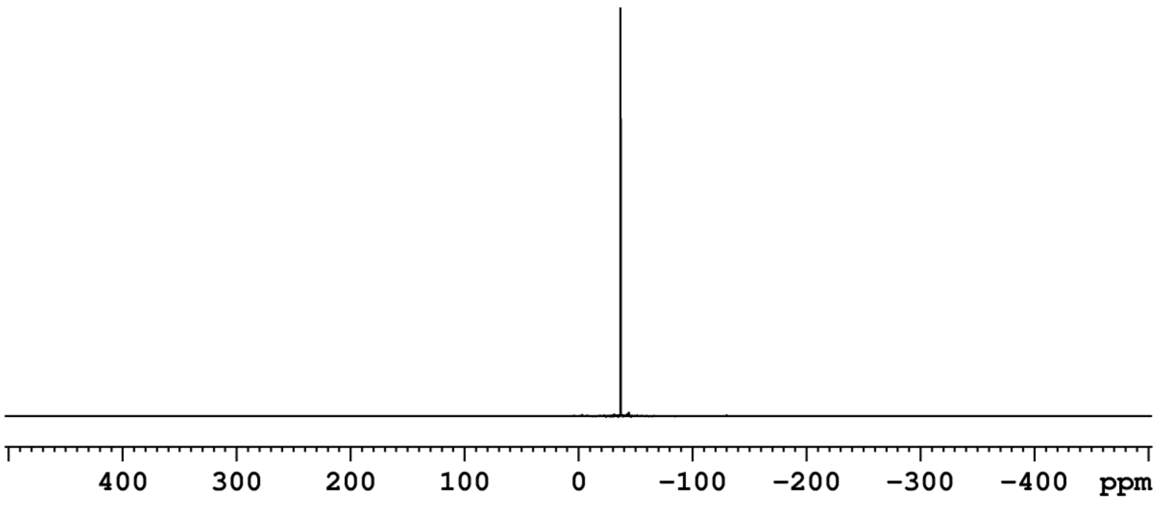

Figure S22. $^{31}P\{^{1}H\}$ NMR spectrum of **5** in CD_2Cl_2.

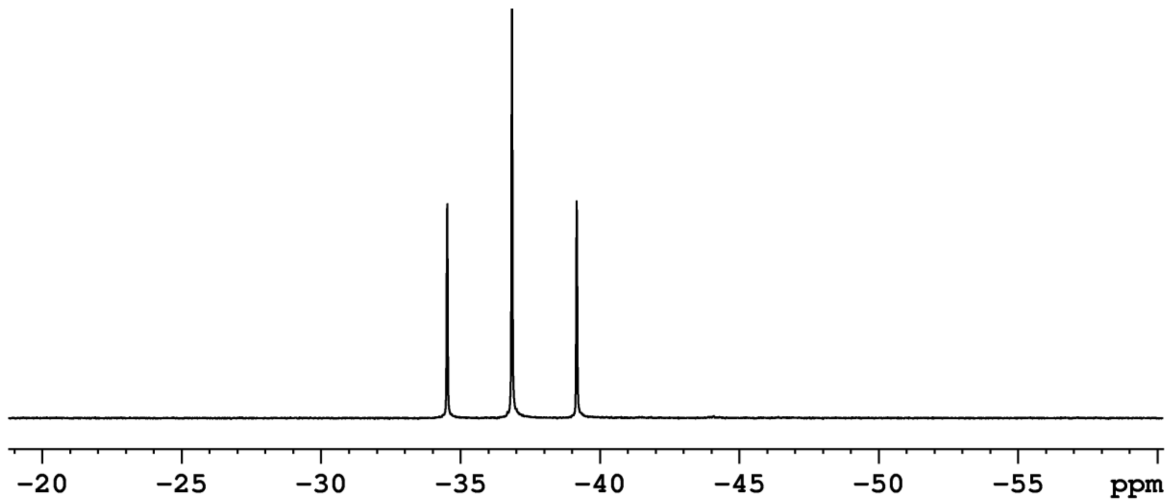

Figure S23. ^{31}P NMR spectrum of **5** in CD_2Cl_2.

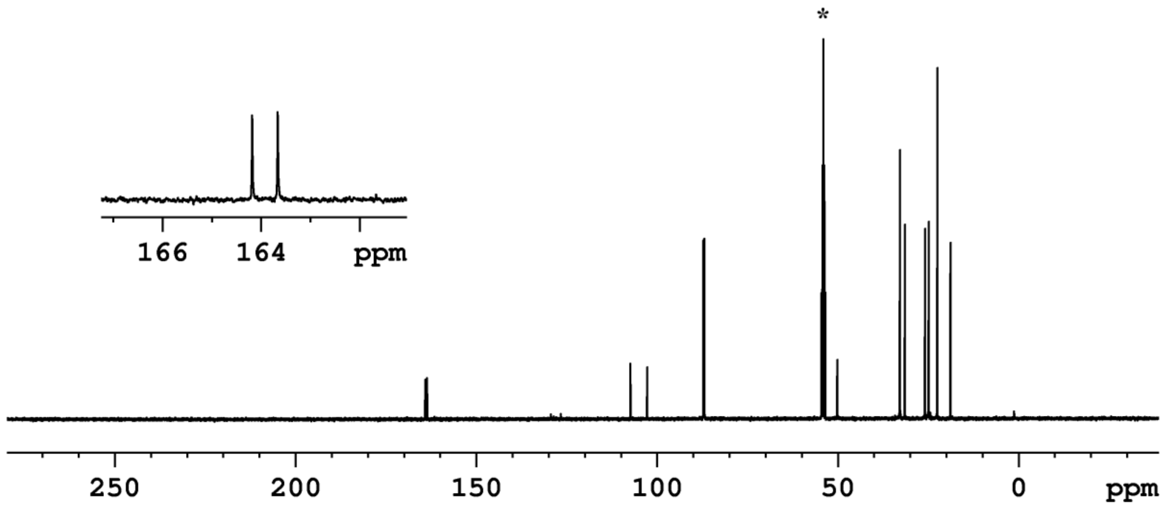

Figure S24. $^{13}C\{^1H\}$ NMR spectrum of **5** in CD_2Cl_2. Resonance marked with * is due to solvent. Inset shows zoomed region of the carbonyl.

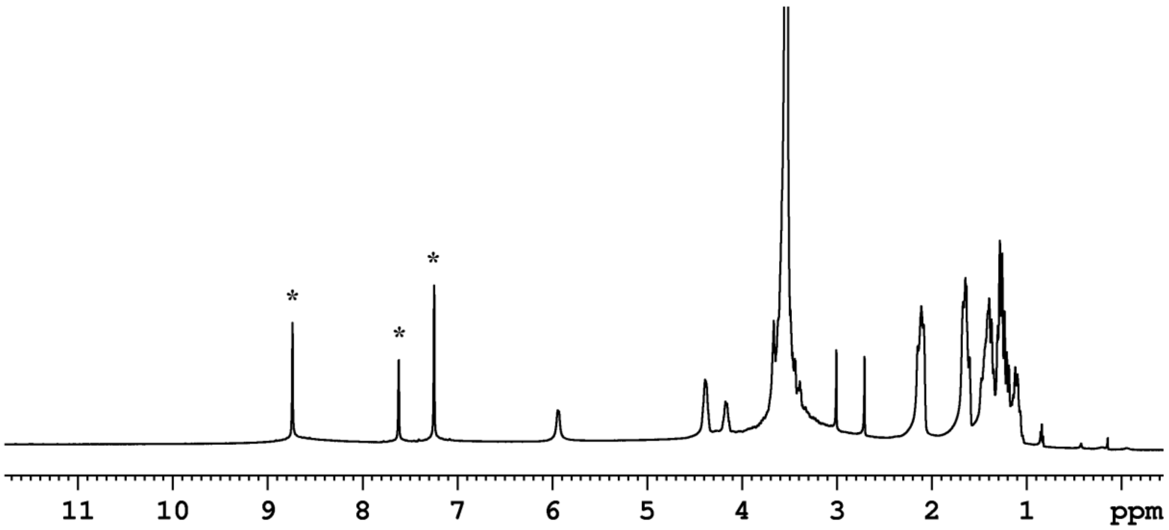

Figure S25. ^{1}H NMR spectrum of [K(18-crown-6)][**6**] in [D_5]-pyridine. Resonances marked with * are due to solvent.

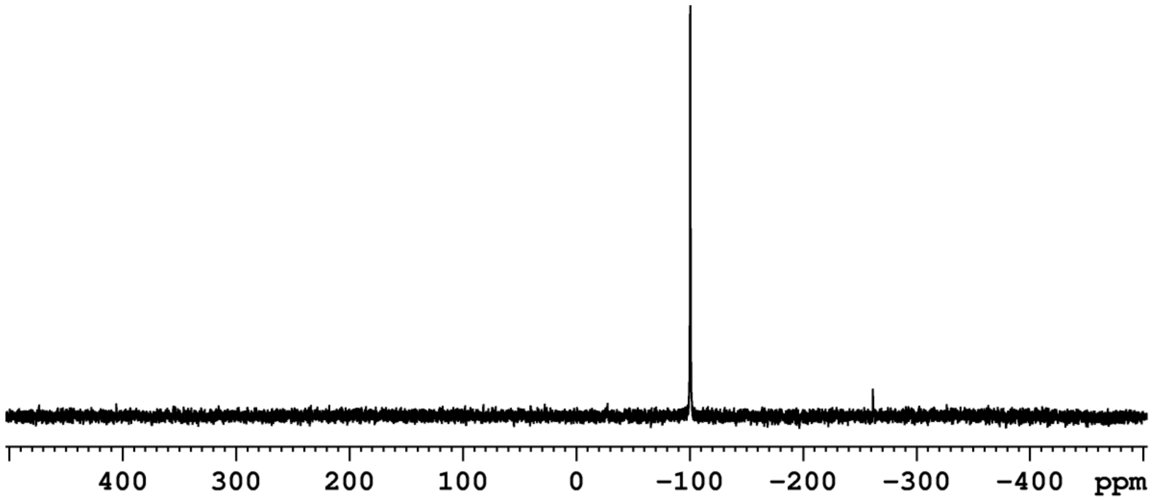

Figure S26. ^{31}P{^{1}H} NMR spectrum of [K(18-crown-6)][**6**] in [D_5]-pyridine.

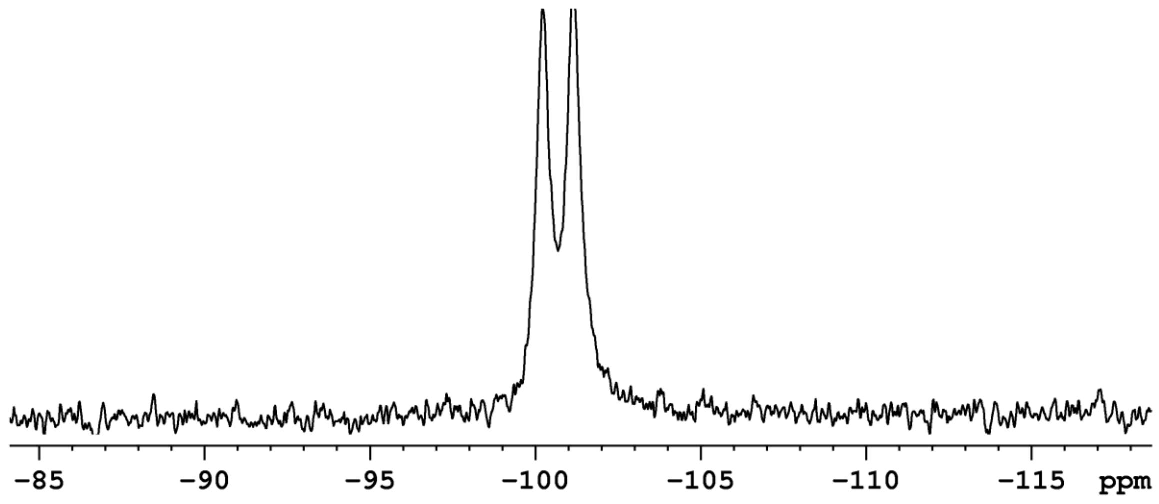

Figure S27. ^{31}P NMR spectrum of [K(18-crown-6)][**6**] in [D_5]-pyridine.

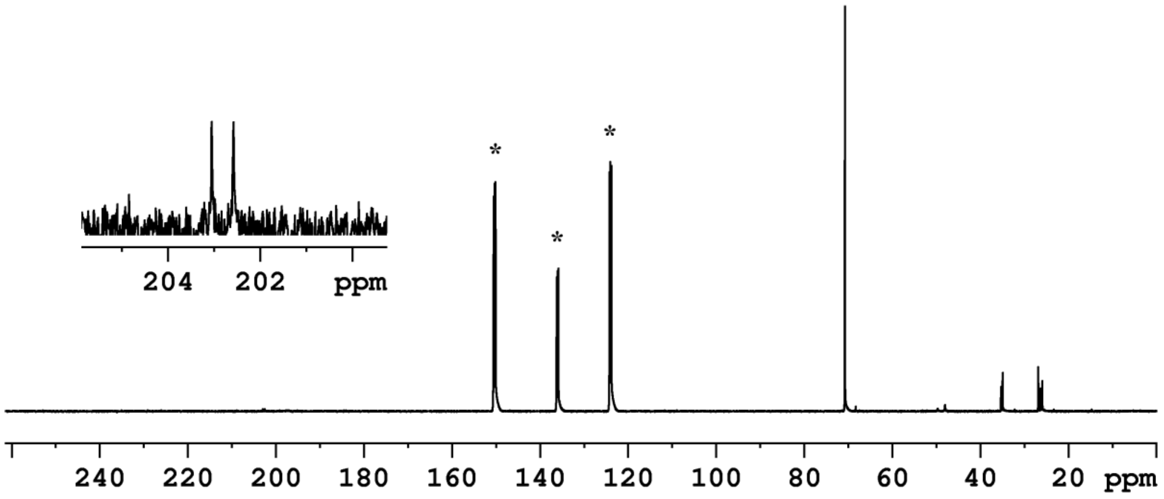

Figure S28. $^{13}C\{^1H\}$ NMR spectrum of [K(18-crown-6)][**6**] in [D_5]-pyridine. Resonances marked with * are due to solvent. Inset shows zoomed region of carbonyl.

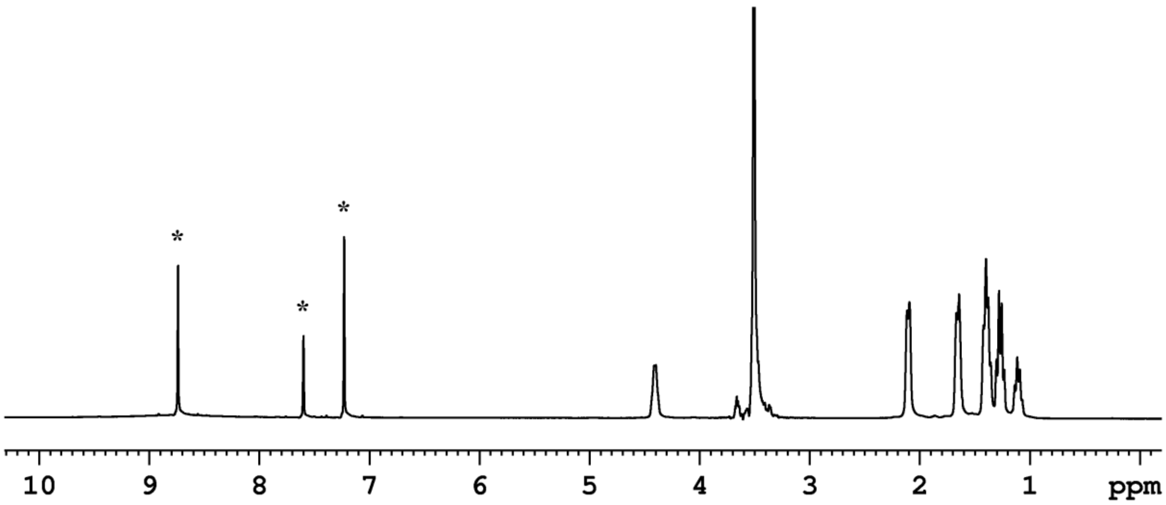

Figure S29. ^{1}H NMR spectrum of [K(18-crown-6)][**7**] in [D_5]-pyridine. Resonances marked with * are due to solvent.

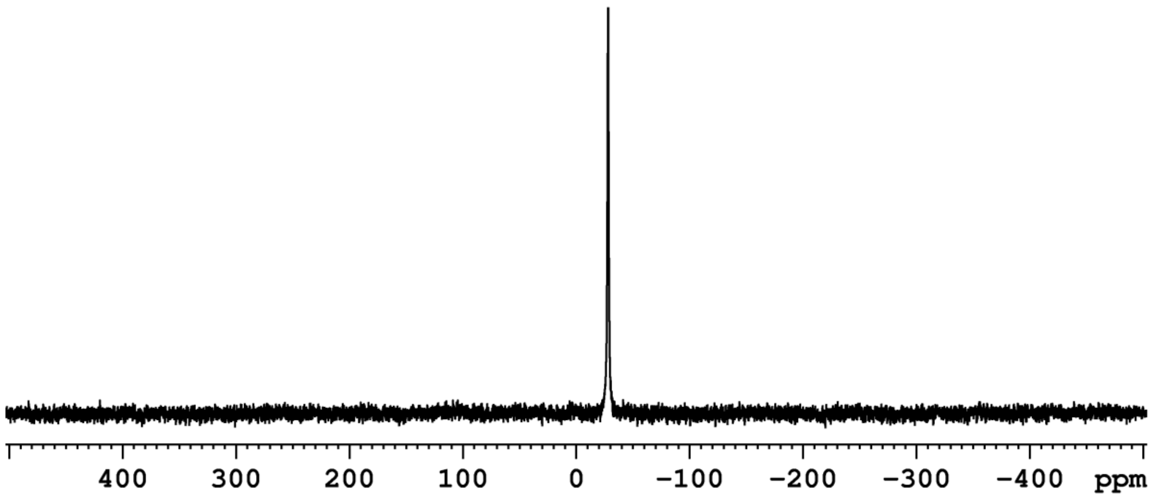

Figure S30. ^{31}P NMR spectrum of [K(18-crown-6)][**7**] in [D_5]-pyridine.

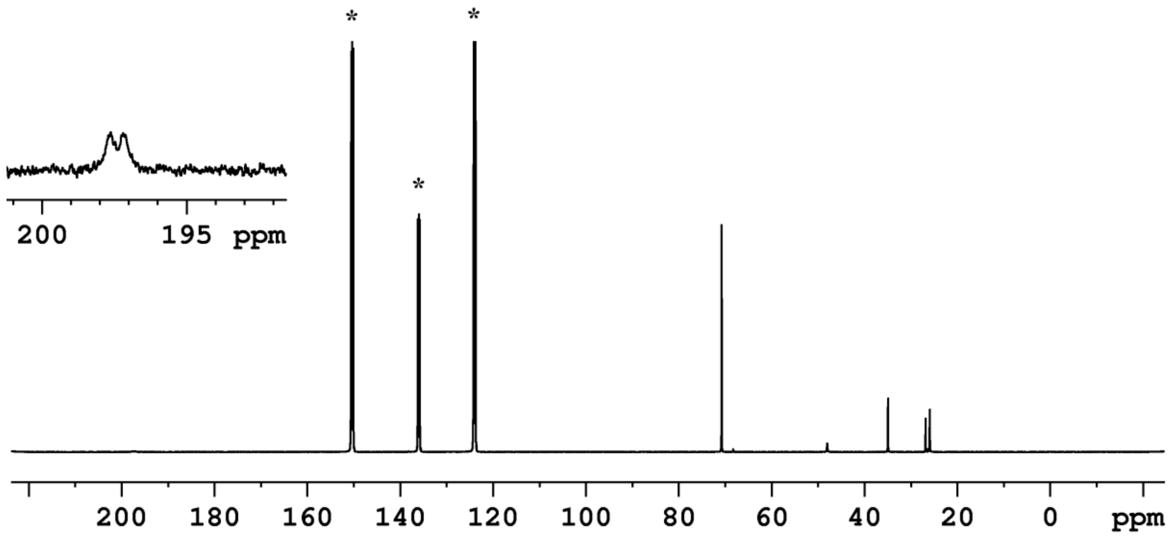

Figure S31. $^{13}C\{^{1}H\}$ NMR spectrum of [K(18-crown-6)][**7**] in [D$_5$]-pyridine. Resonances marked with * are due to solvent. Inset shows zoomed region of the carbonyl.

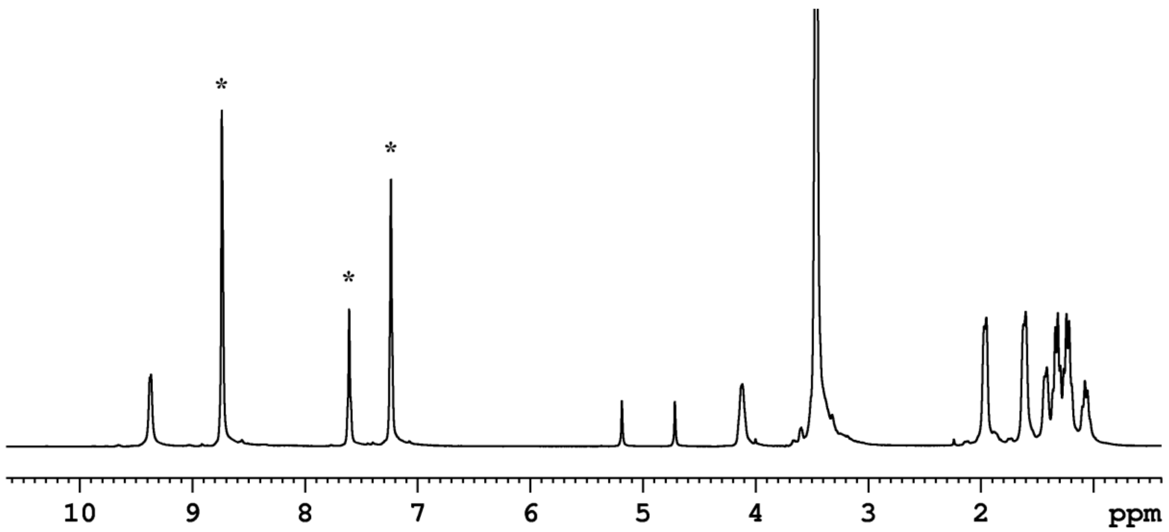

Figure S32. ^{1}H NMR spectrum of **8** in [D$_5$]-pyridine. Resonances marked with * are due to solvent.

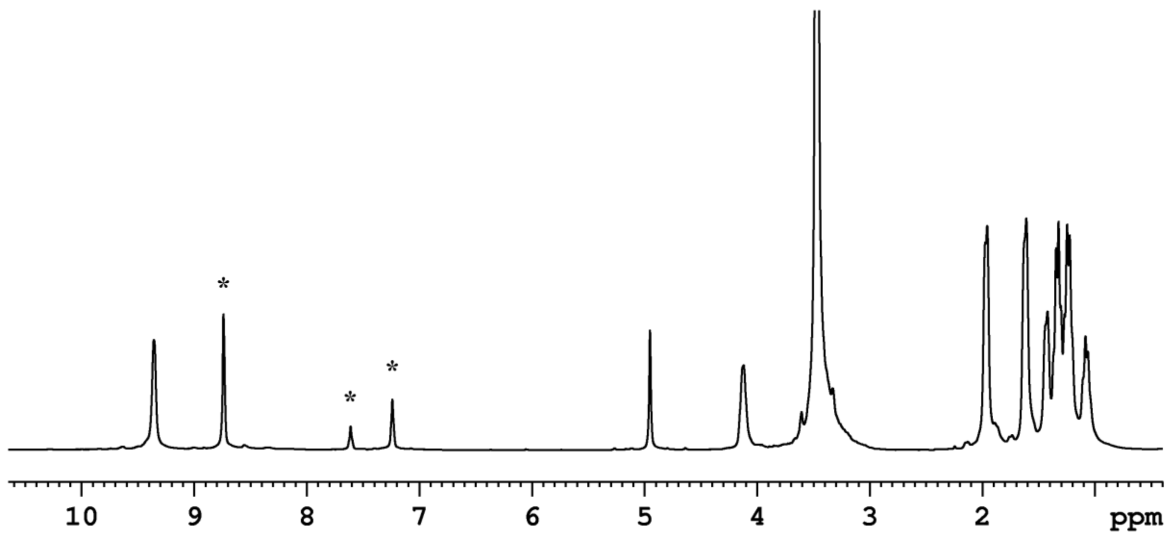

Figure S33. $^{1}H\{^{31}P\}$ NMR spectrum of **8** in [D_5]-pyridine. Resonances marked with * are due to solvent.

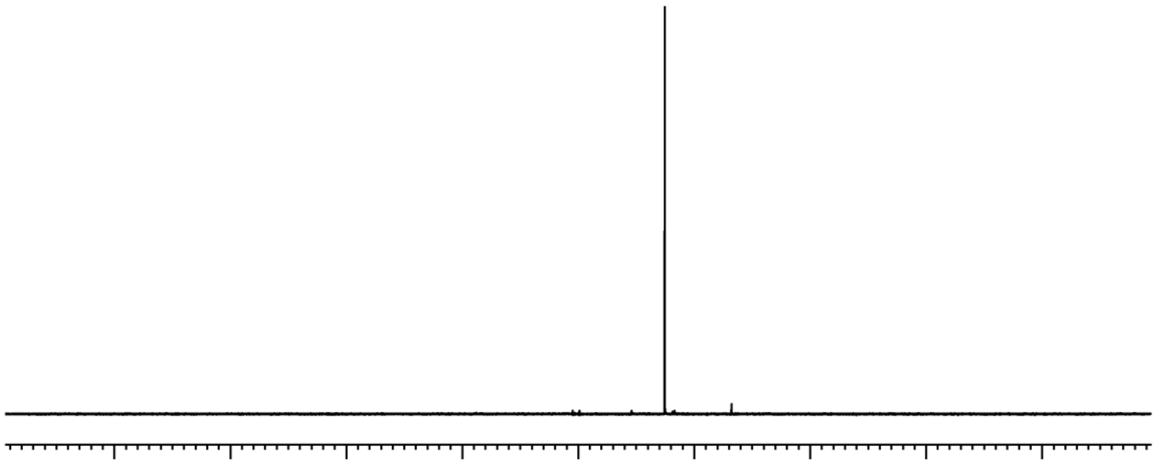

Figure S34. $^{31}P\{^{1}H\}$ NMR spectrum of **8** in [D_5]-pyridine.

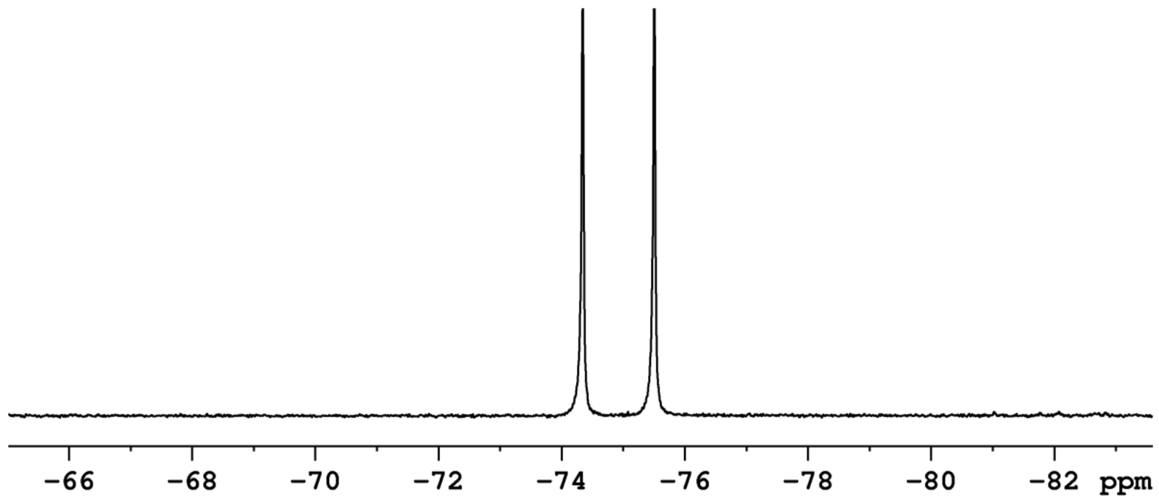

Figure S35. ^{31}P NMR spectrum of **8** in [D_5]-pyridine.

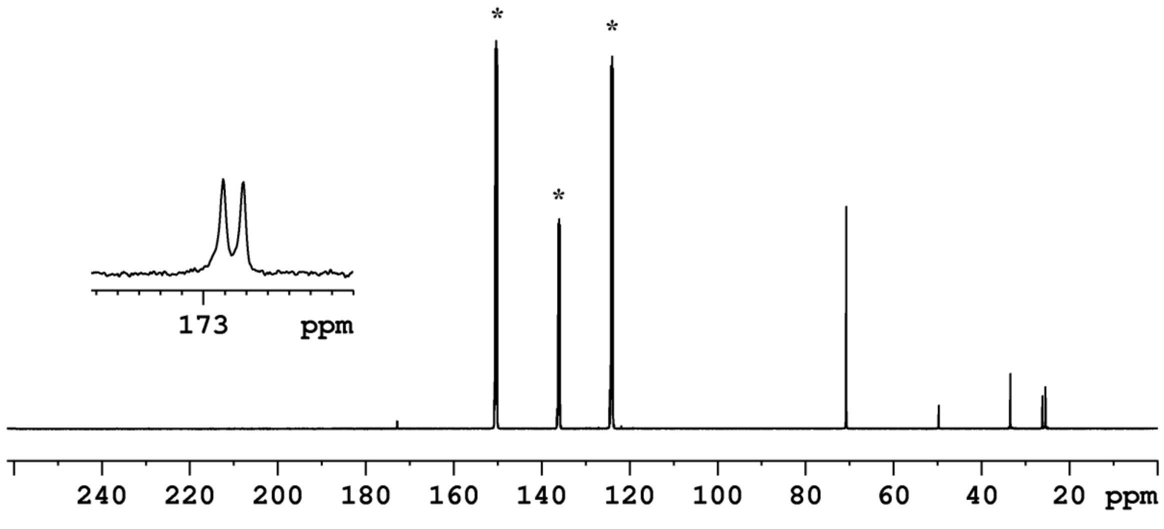

Figure S36. $^{13}C\{^1H\}$ NMR spectrum of **8** in [D_5]-pyridine. Resonances marked with * are due to solvent. Inset shows zoomed region of the carbonyl.

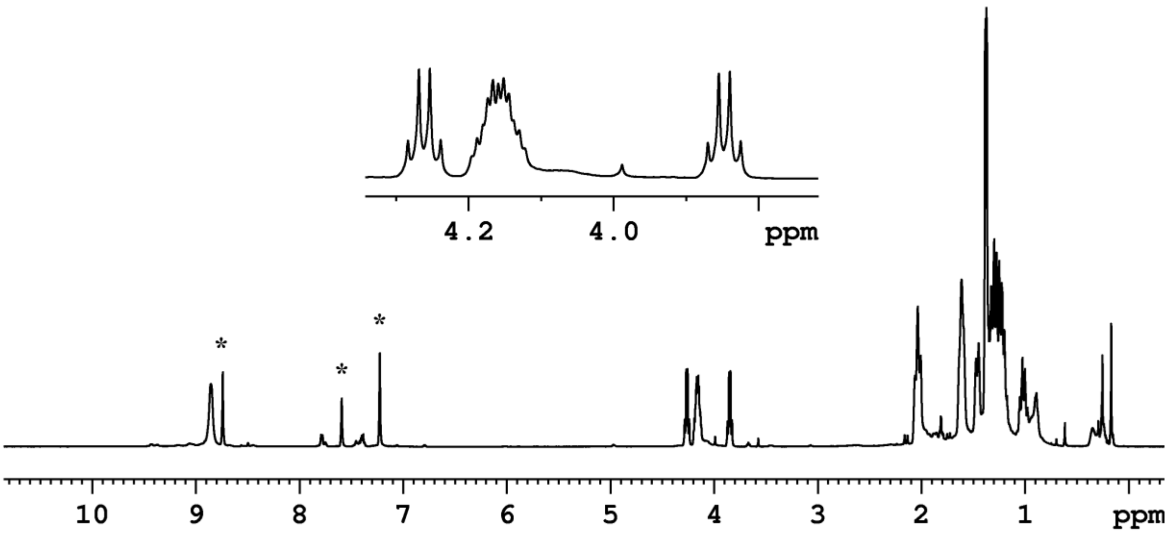

Figure S37. ^{1}H NMR spectrum of **9** in [D_5]-pyridine. Resonances marked with * are due to solvent. Inset shows doublet of quartets attributed to P–H proton.

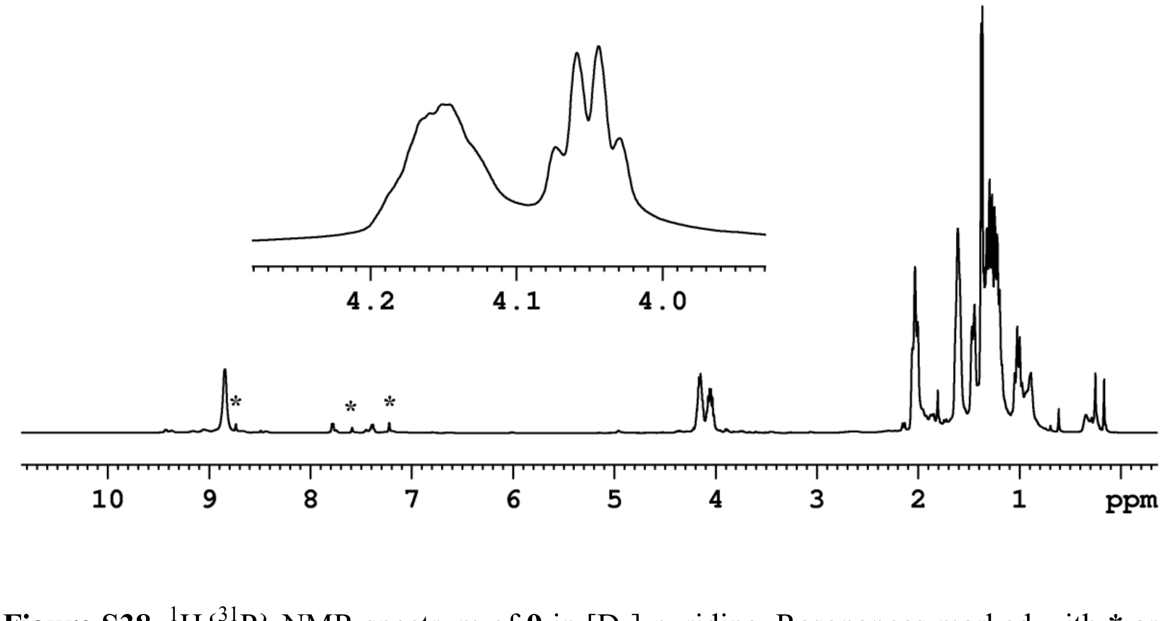

Figure S38. $^{1}H\{^{31}P\}$ NMR spectrum of **9** in [D_5]-pyridine. Resonances marked with * are due to solvent. Inset shows quartet from P–H proton, no longer coupling to the P.

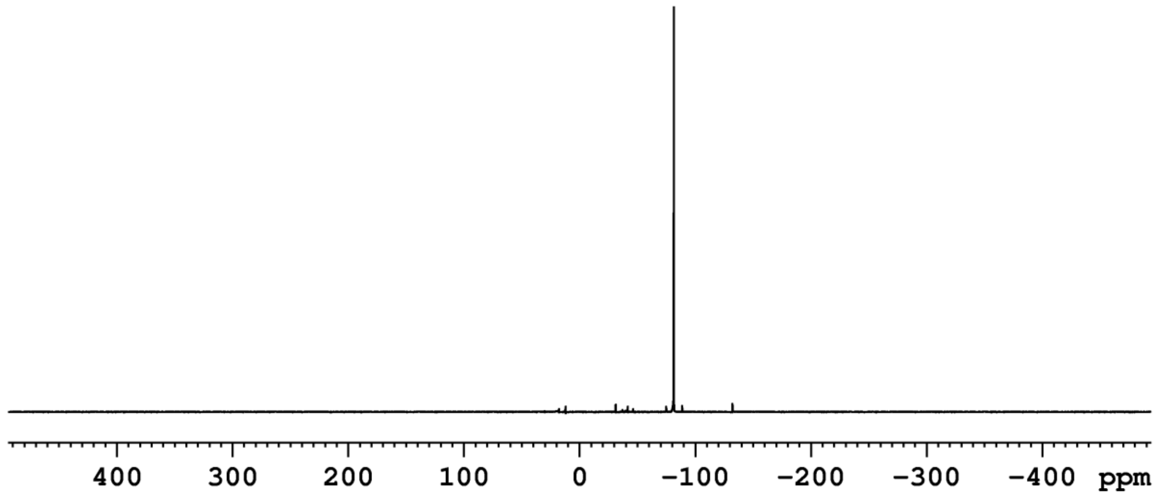

Figure S39. $^{31}P\{^{1}H\}$ NMR spectrum of **9** in [D_5]-pyridine.

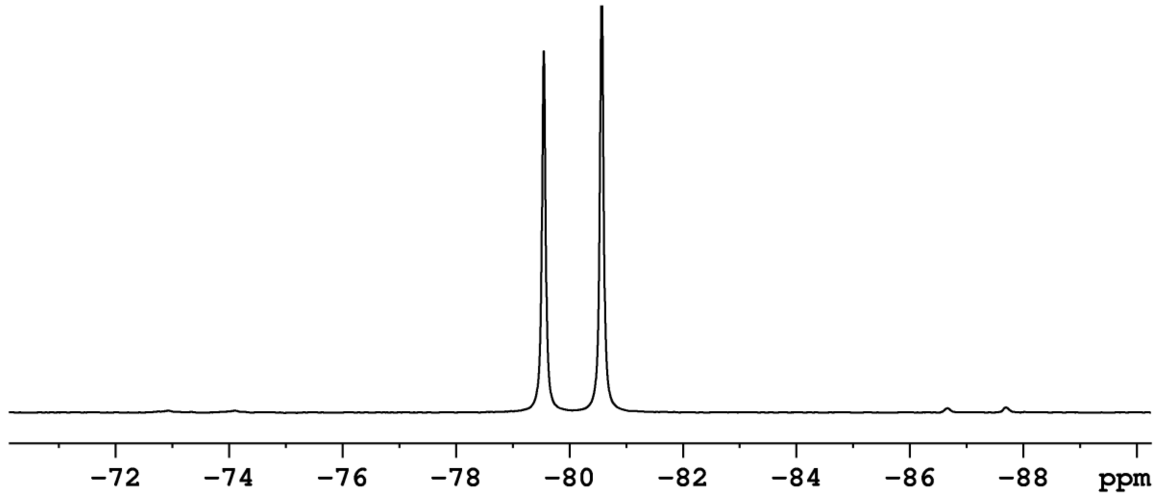

Figure S40. ^{31}P NMR spectrum of **9** in [D_5]-pyridine.

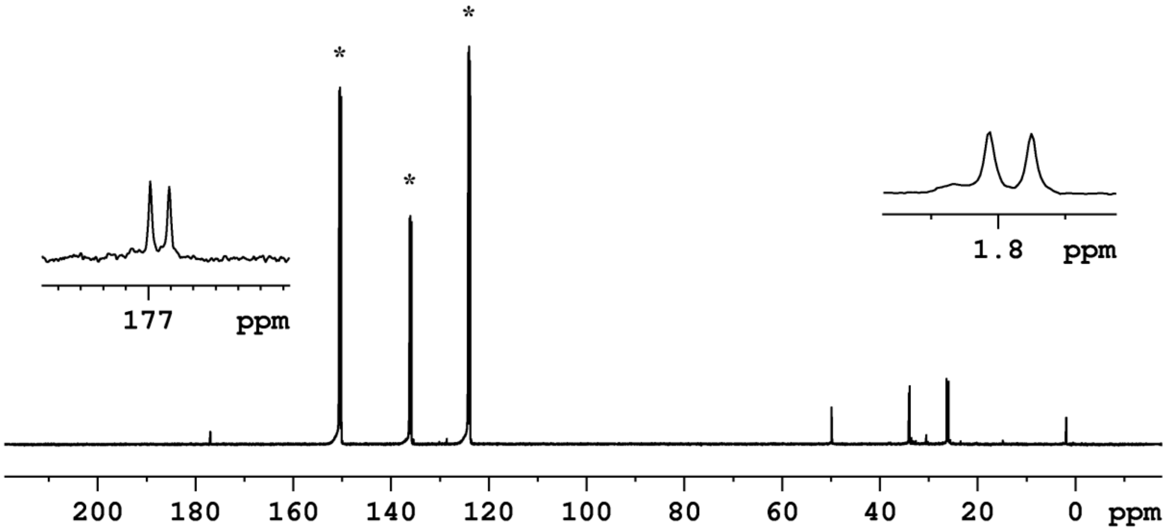

Figure S41. $^{13}C\{^{1}H\}$ NMR spectrum of **9** in [D$_5$]-pyridine. Resonances marked with * are due to solvent. Insets show zoomed regions of the carbonyl and the methyl groups.

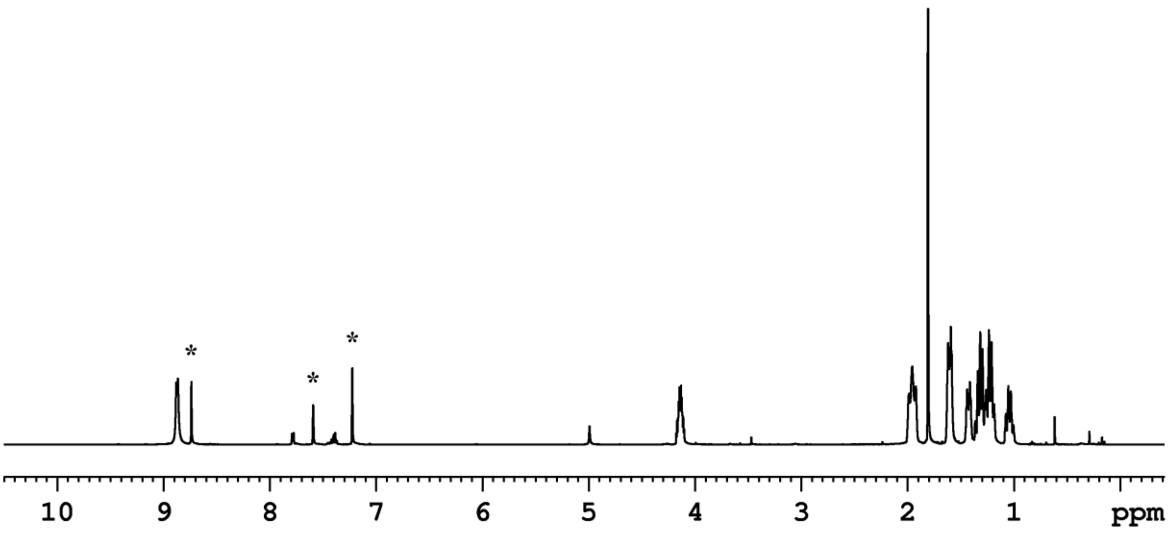

Figure S42. ^{1}H NMR spectrum of **10** in [D$_5$]-pyridine. Resonances marked with * are due to solvent.

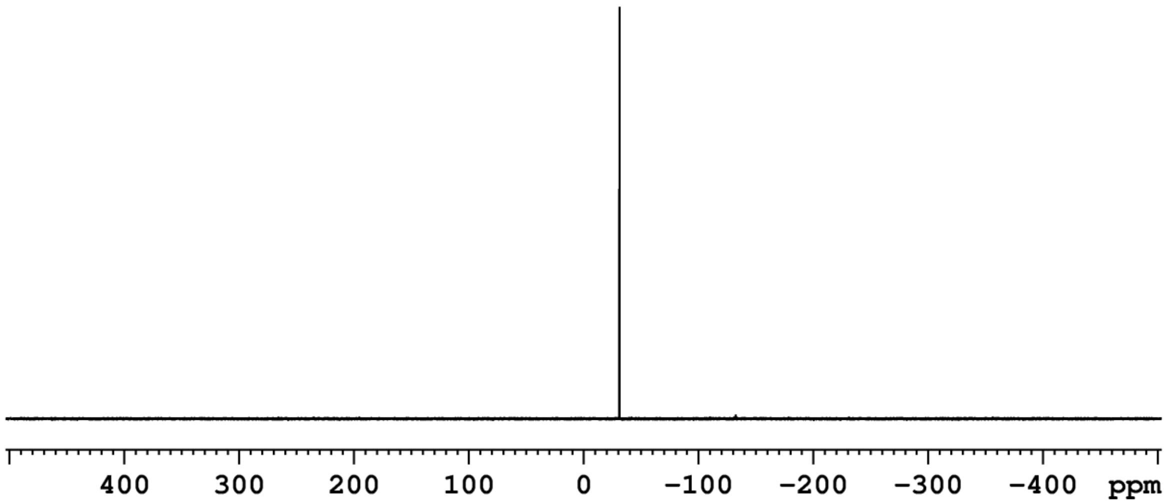

Figure S43. $^{31}P\{^{1}H\}$ NMR spectrum of **10** in [D$_5$]-pyridine.

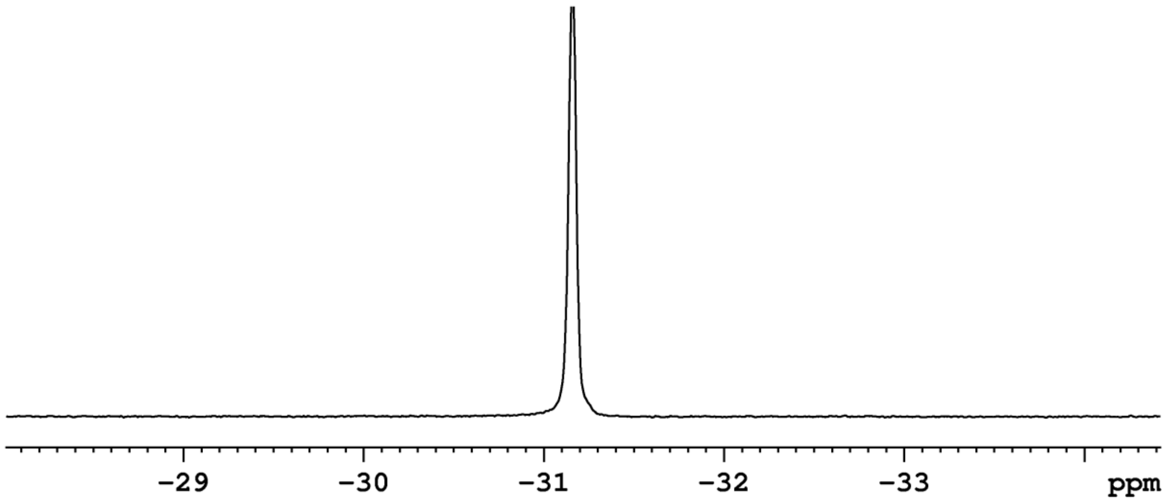

Figure S44. ^{31}P NMR spectrum of **10** in [D$_5$]-pyridine. Do not resolve the expected quartet pattern from the coupling to the methyl group, but the resonance is broader than in the $^{31}P\{^{1}H\}$ NMR spectrum in S43.

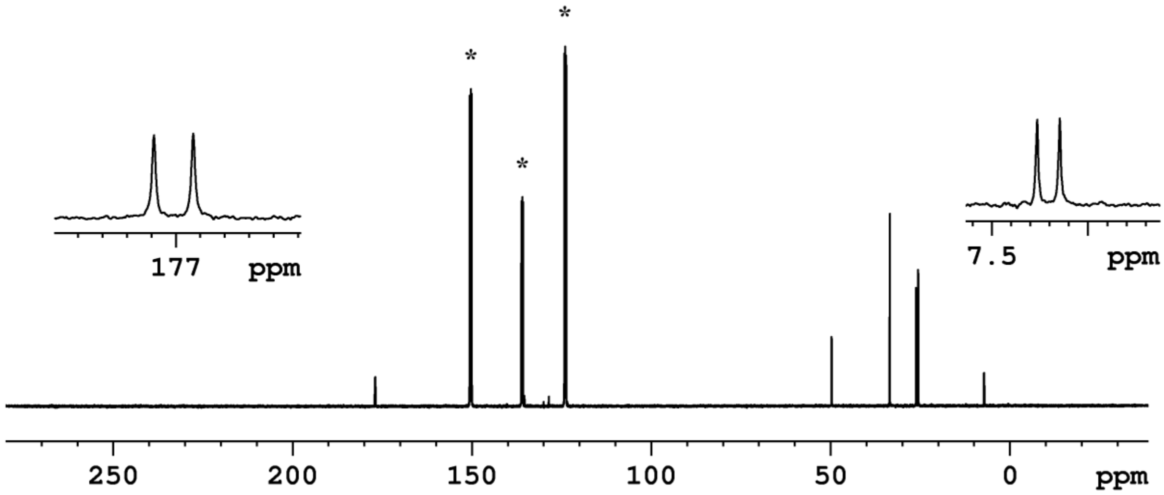

Figure S45. $^{13}C\{^{1}H\}$ NMR spectrum of **10** in [D_5]-pyridine. Resonances marked with * are due to solvent. Insets show zoomed regions of the carbonyl and the methyl groups.

5. MS spectra

5.1 FI-MS

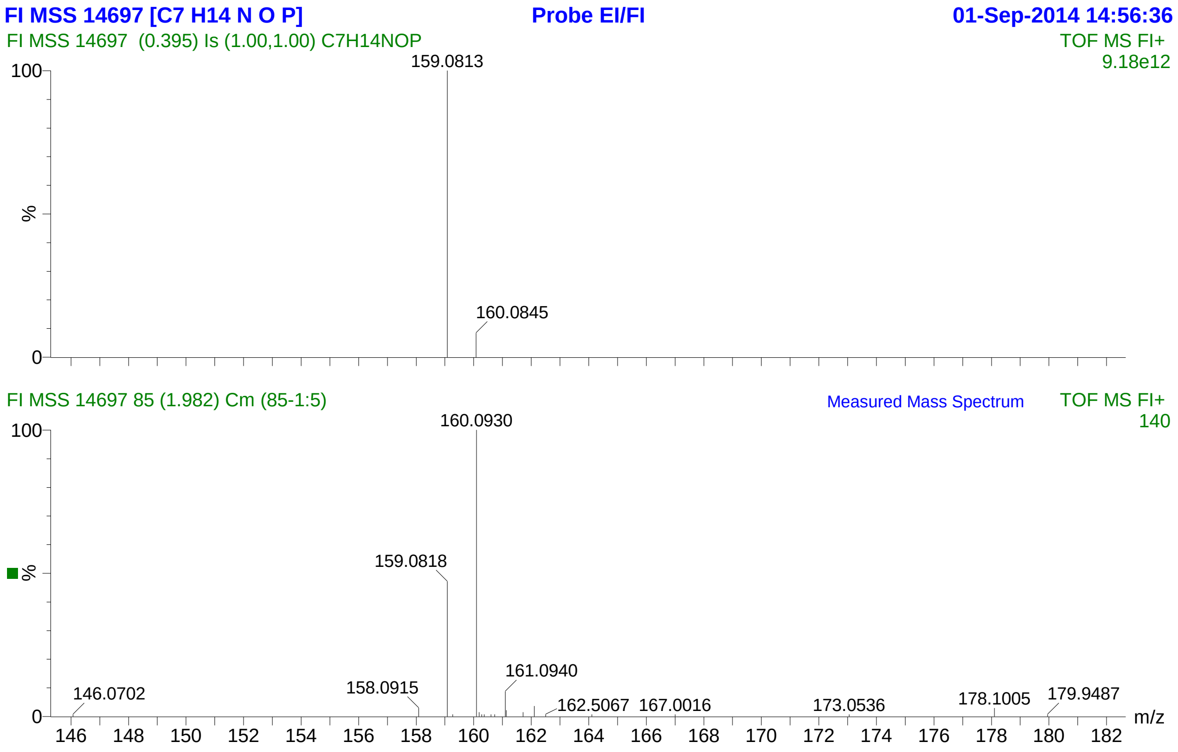

Figure S46. FI-MS of a solid sample of **2**. Theoretical isotope model above, measured mass spectrum below.

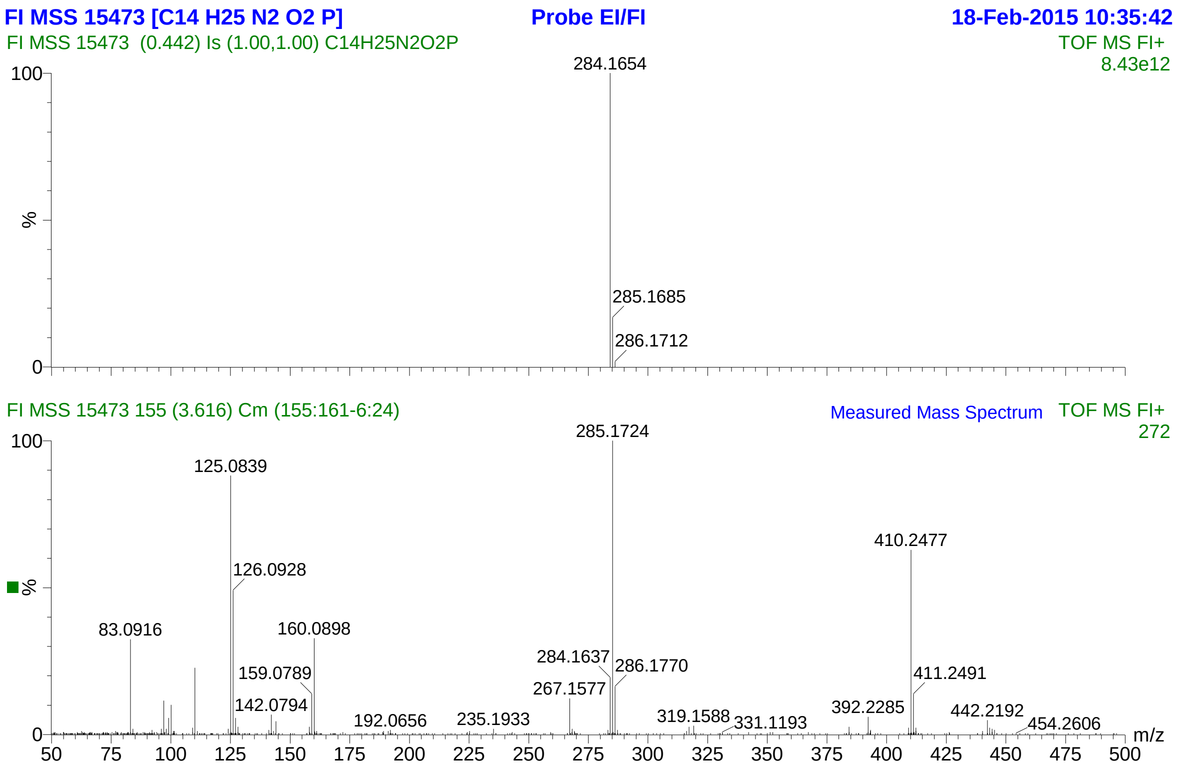

Figure S47. FI-MS of a solid sample of **8**. Theoretical isotope model above, measured mass spectrum below. Presence of other mass envelopes explained in experimental section 1.3.8.

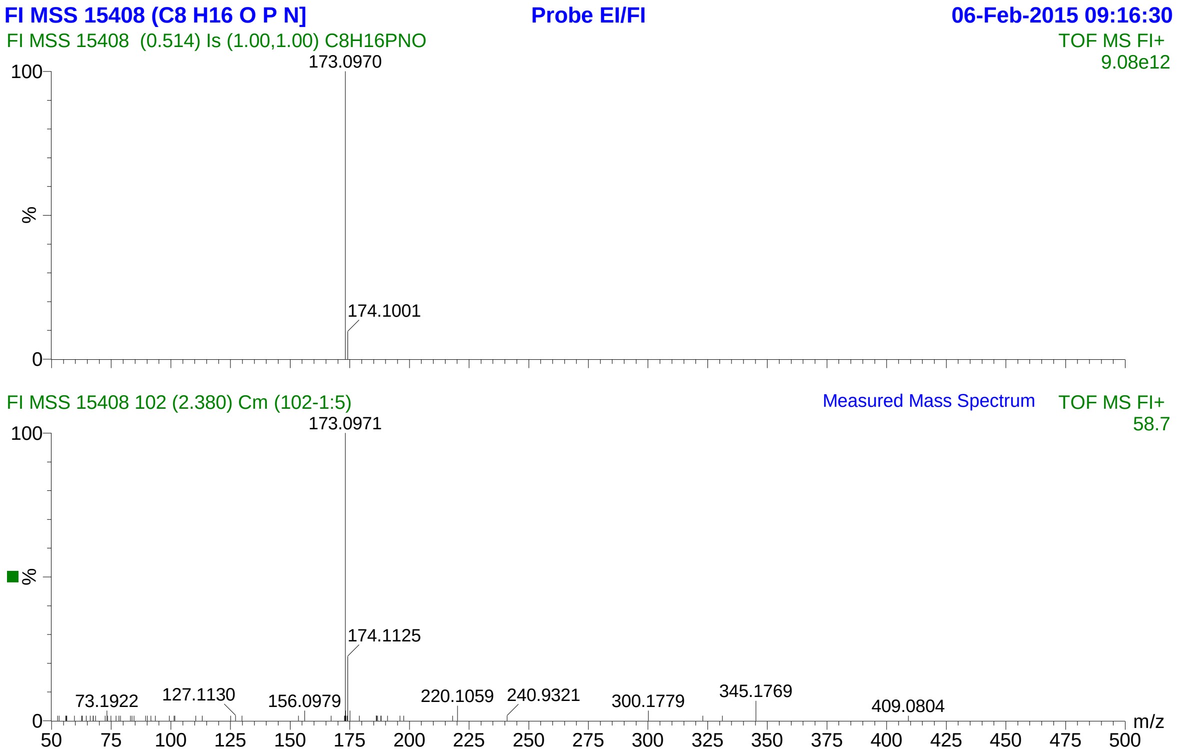

Figure S48. FI-MS of a solid sample of **9**. Theoretical isotope model above, measured mass spectrum below.

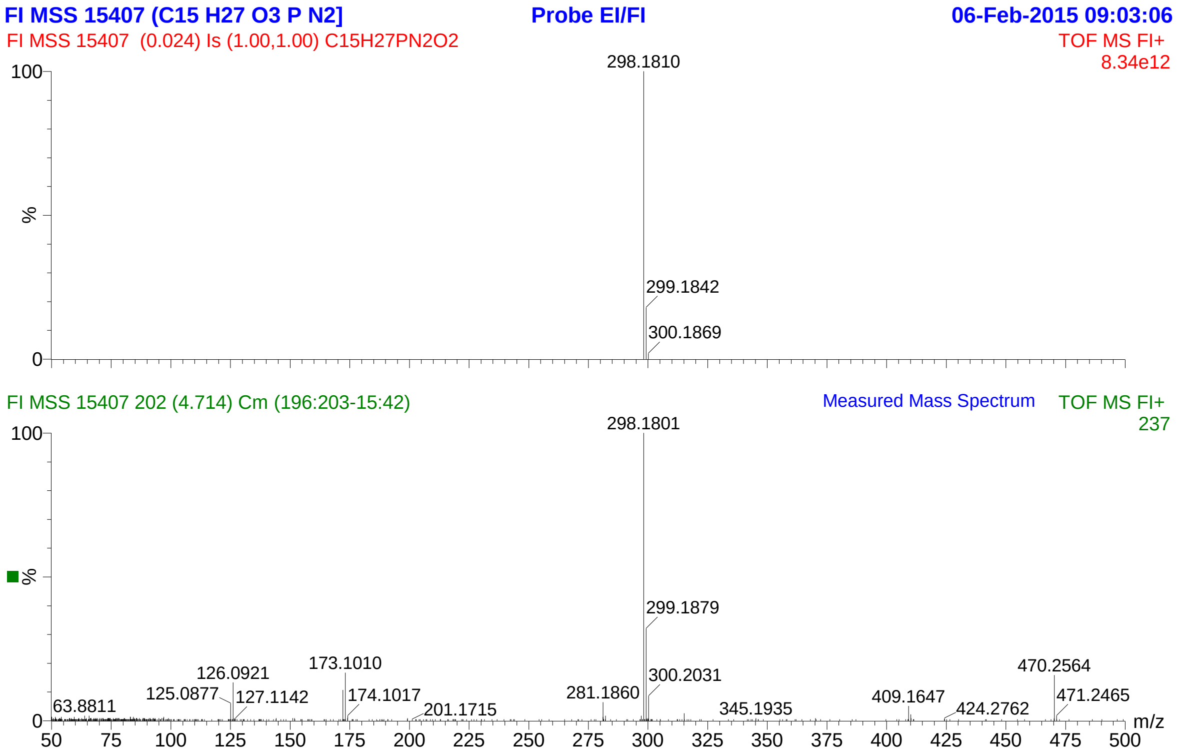

Figure S49. FI-MS of a solid sample of **10**. Theoretical isotope model above, measured mass spectrum below.

5.2 EI-MS

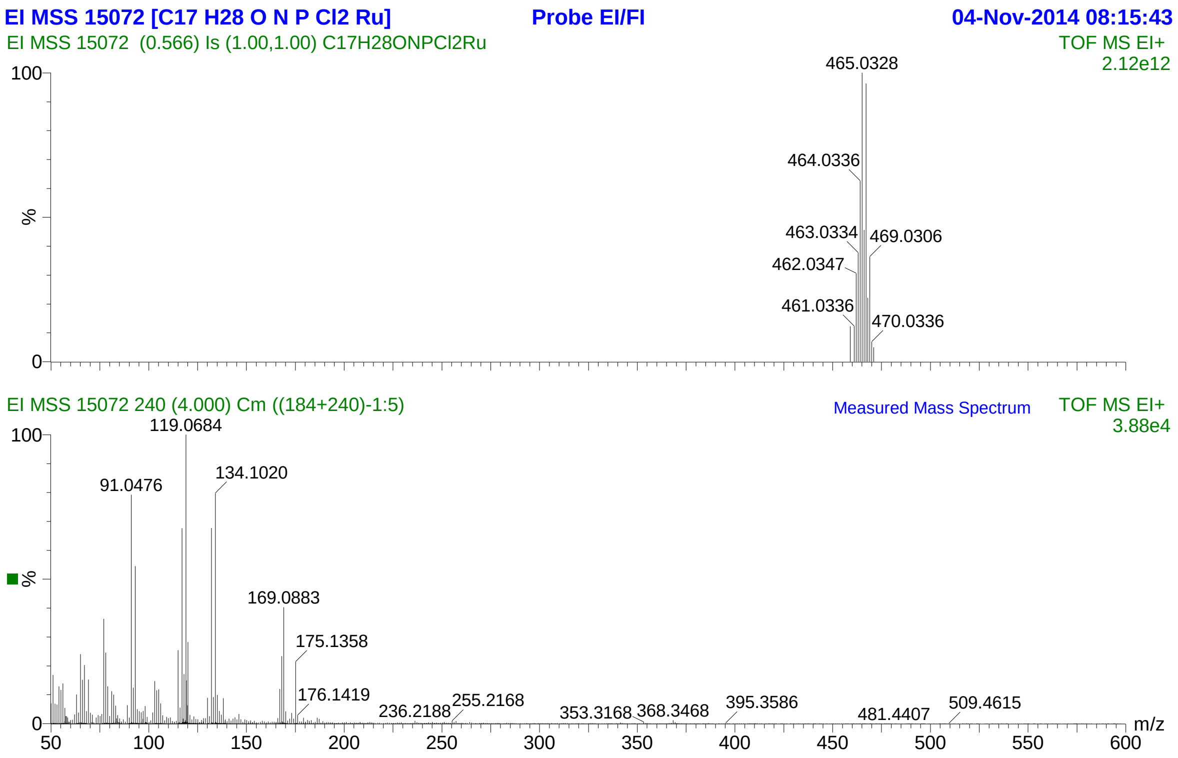

Figure S50. EI-MS of a solid sample of **5**. Theoretical isotope model above, measured mass spectrum below. Note complete fragmentation of product to only observe *p*-cymene and fragments thereof.

5.3 ESI-MS

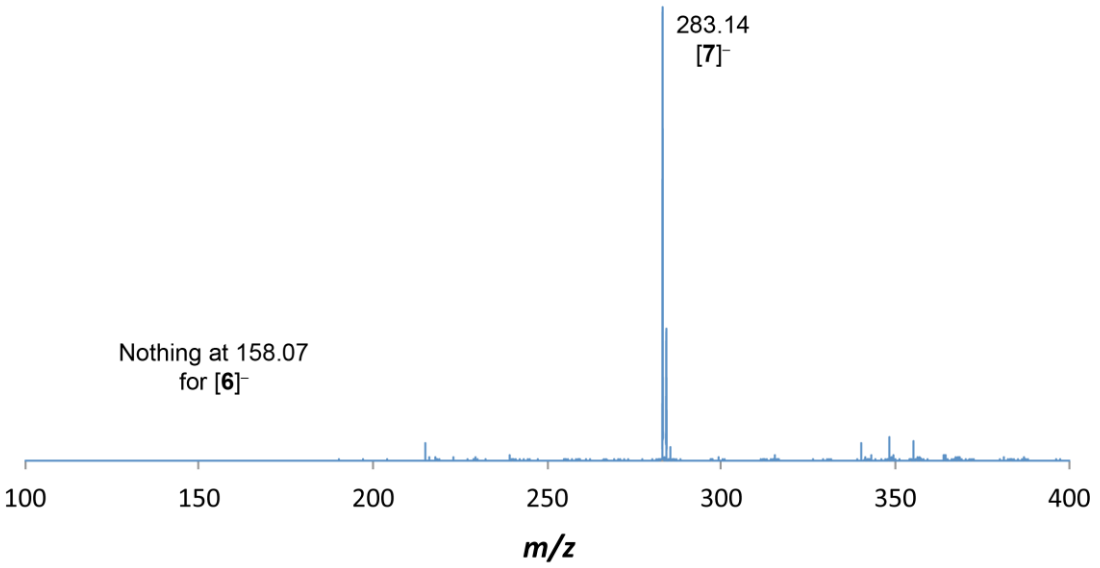

Figure S51. ESI-MS (negative mode) of a DMF solution of a mixture of [K(18-crown-6)][**6**] and [K(18-crown-6)][**7**]. Note presence of [**7**]$^-$, but absence of [**6**]$^-$ in negative mode.

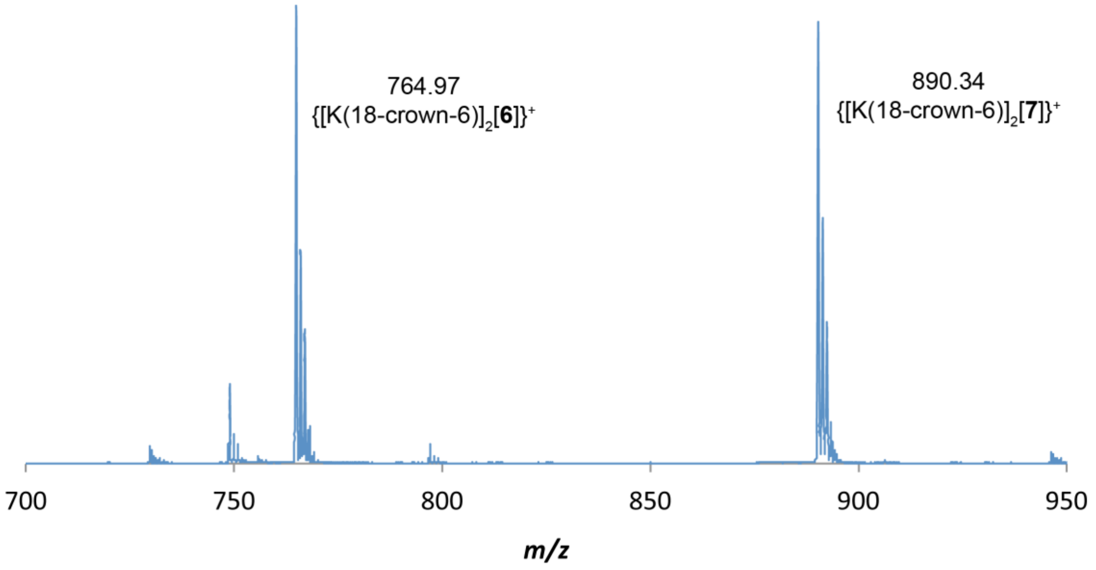

Figure S52. ESI-MS (positive mode) of a DMF solution of a mixture of [K(18-crown-6)][**6**] and [K(18-crown-6)][**7**]. Note presence of both {[K(18-crown-6)]$_2$[**6**]}$^+$ and {[K(18-crown-6)]$_2$[**7**]}$^+$ in positive mode.

6. IR spectra

6.1 Solution phase IR spectra

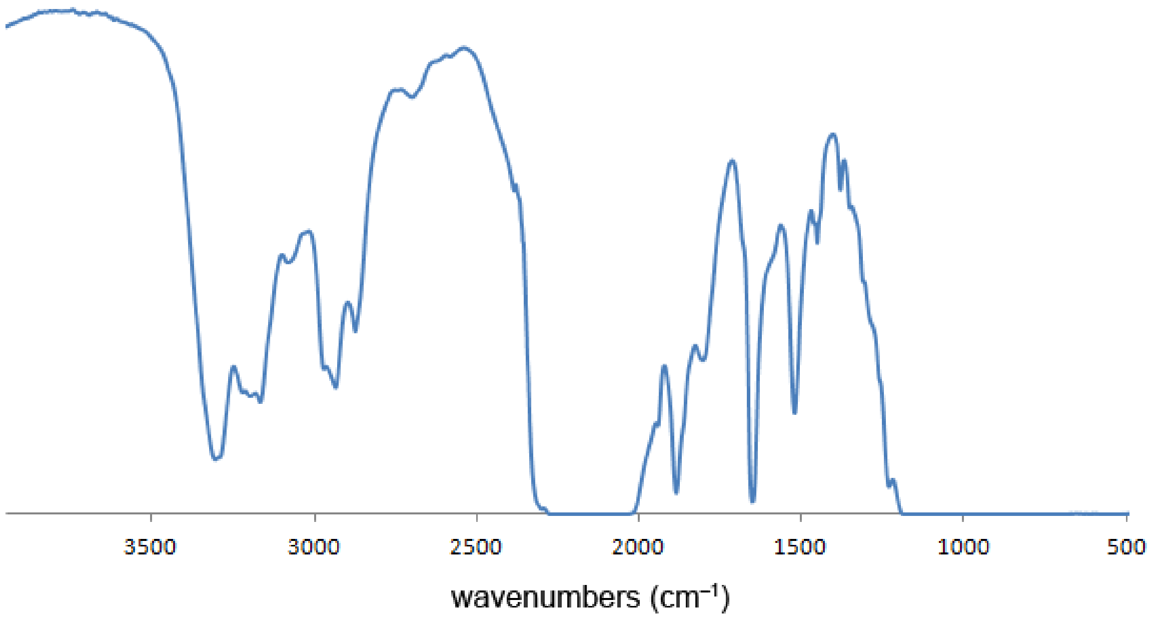

Figure S53. IR spectrum of **1** in [D_8]-THF.

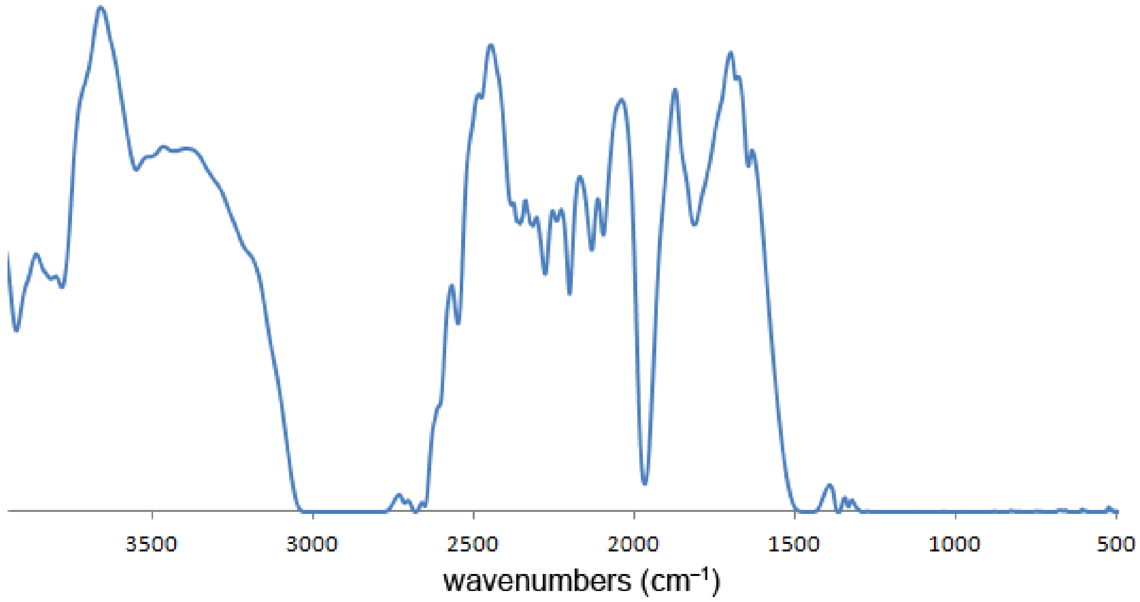

Figure S54. IR spectrum of **2** in THF.

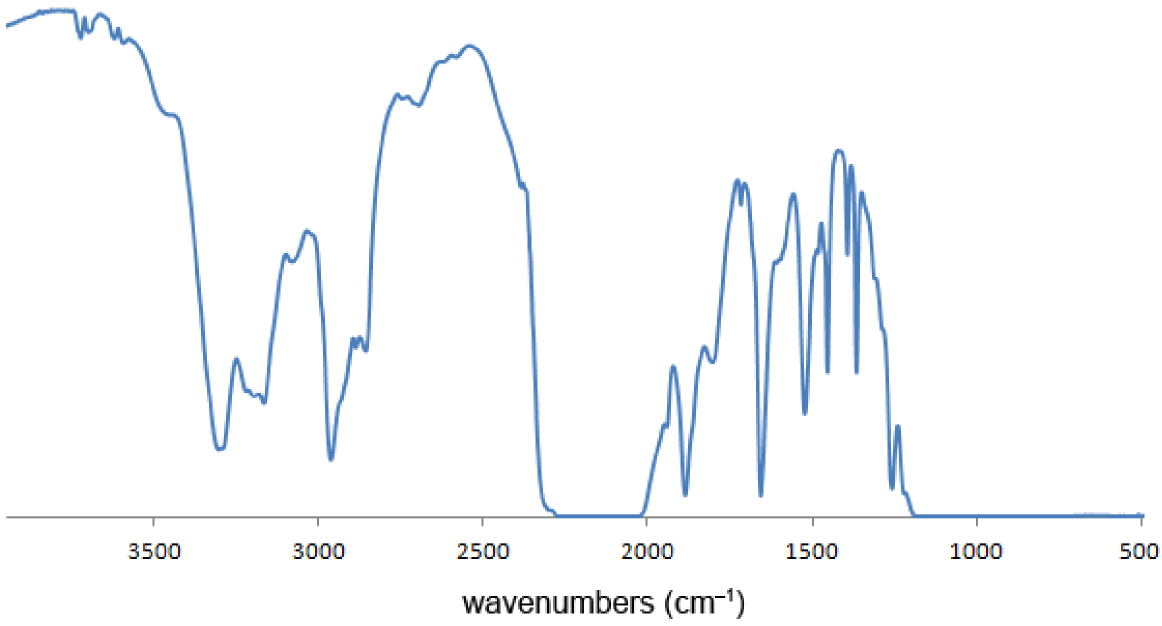

Figure S55. IR spectrum of **3** in [D$_8$]-THF.

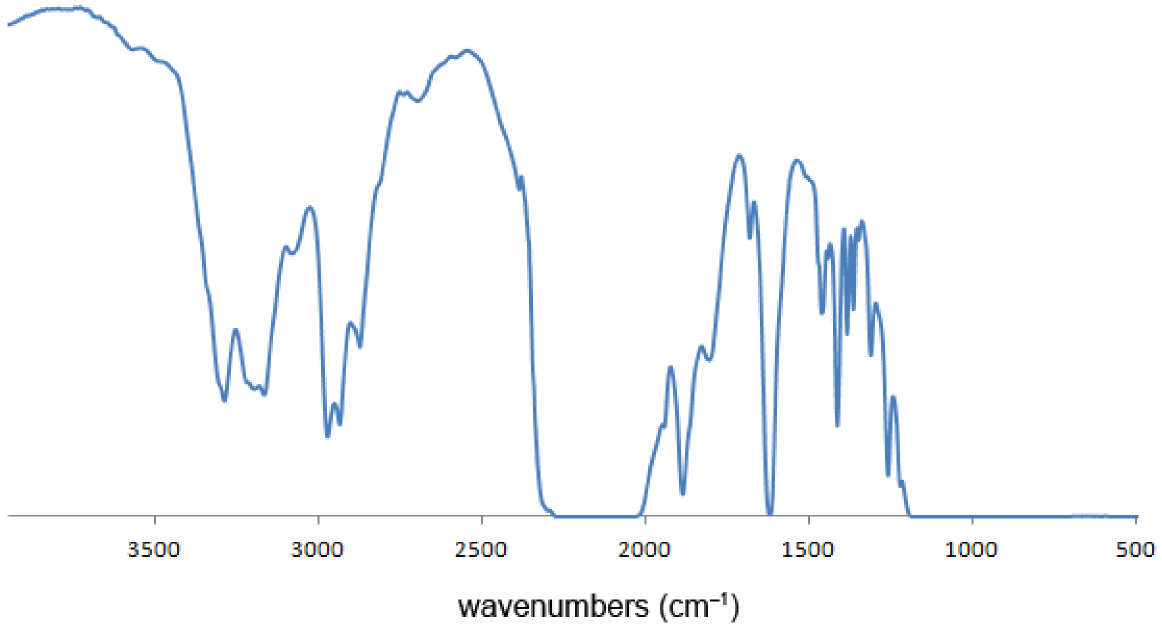

Figure S56. IR spectrum of **4** in [D$_8$]-THF.

6.2 Solid state IR spectra

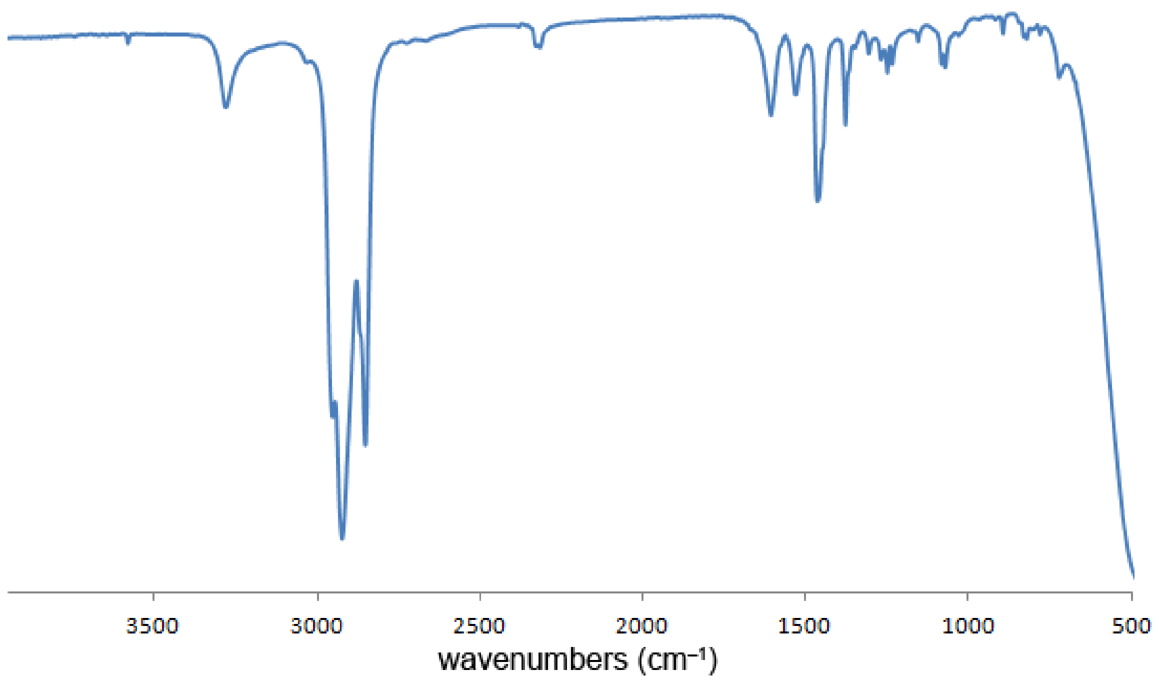

Figure S57. IR spectrum of **2** in a Nujol mull.

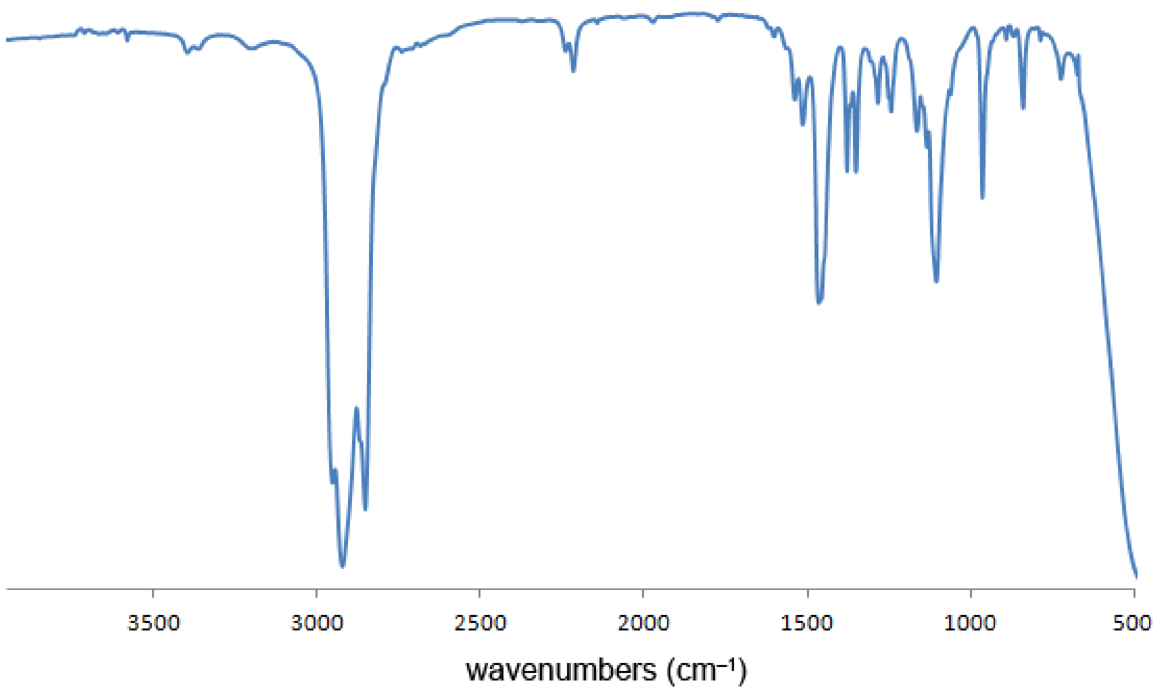

Figure S58. IR spectrum of [K(18-crown-6)][**6**] in a Nujol mull.

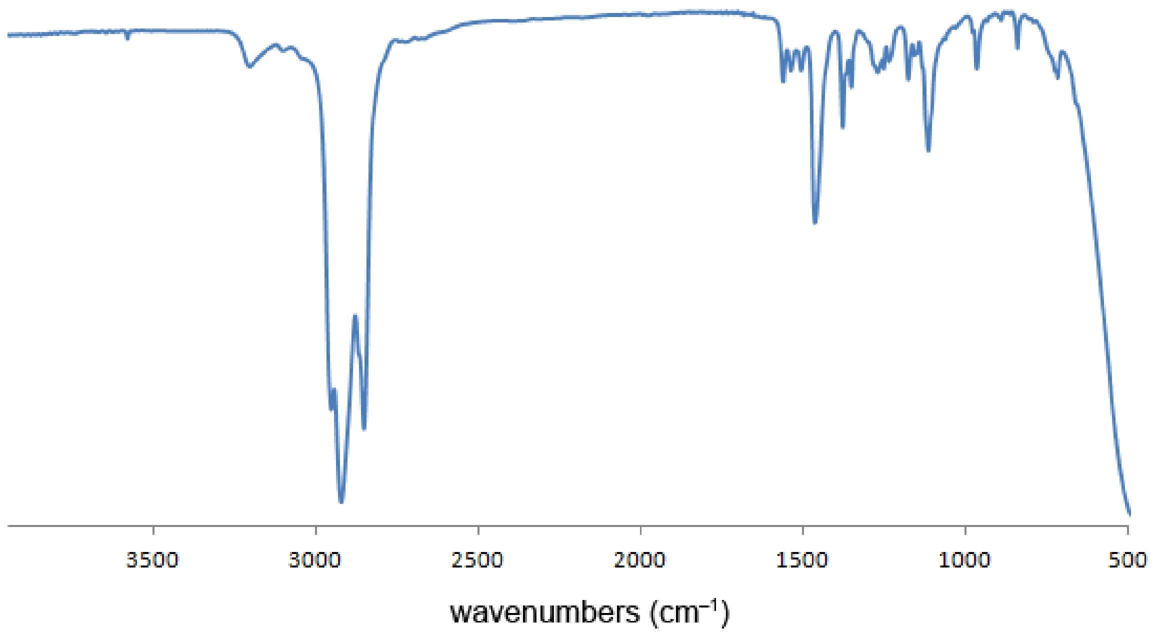

Figure S59. IR spectrum of [K(18-crown-6)][**7**] in a Nujol mull.

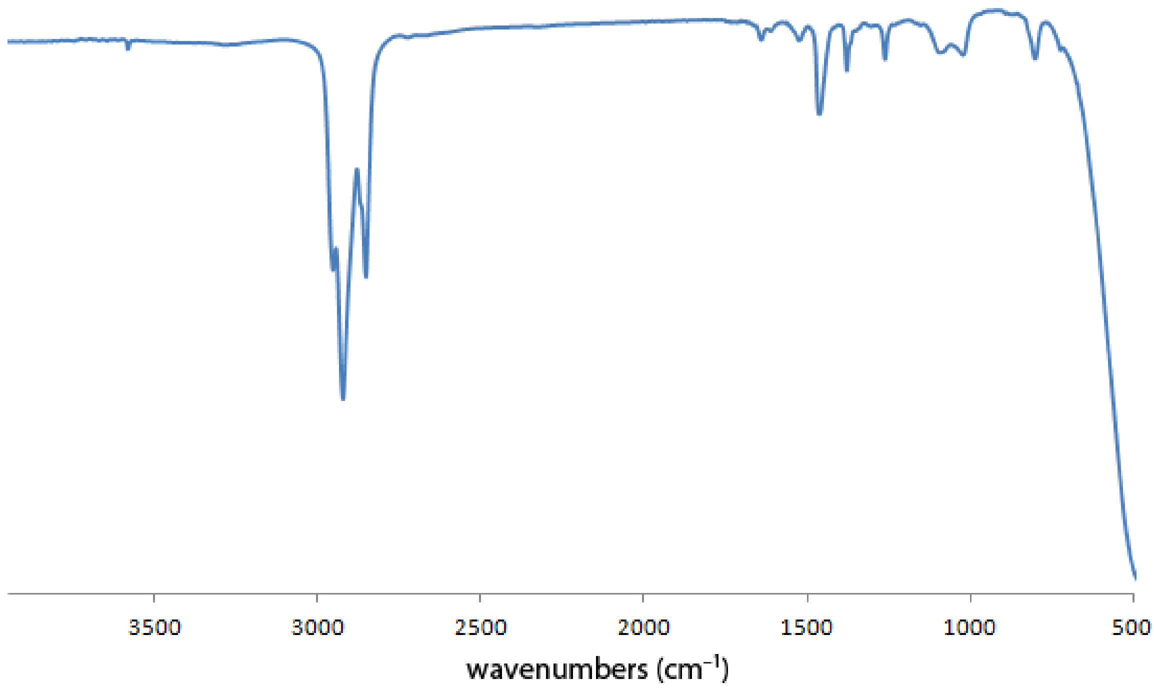

Figure S60. IR spectrum of **8** in a Nujol mull.

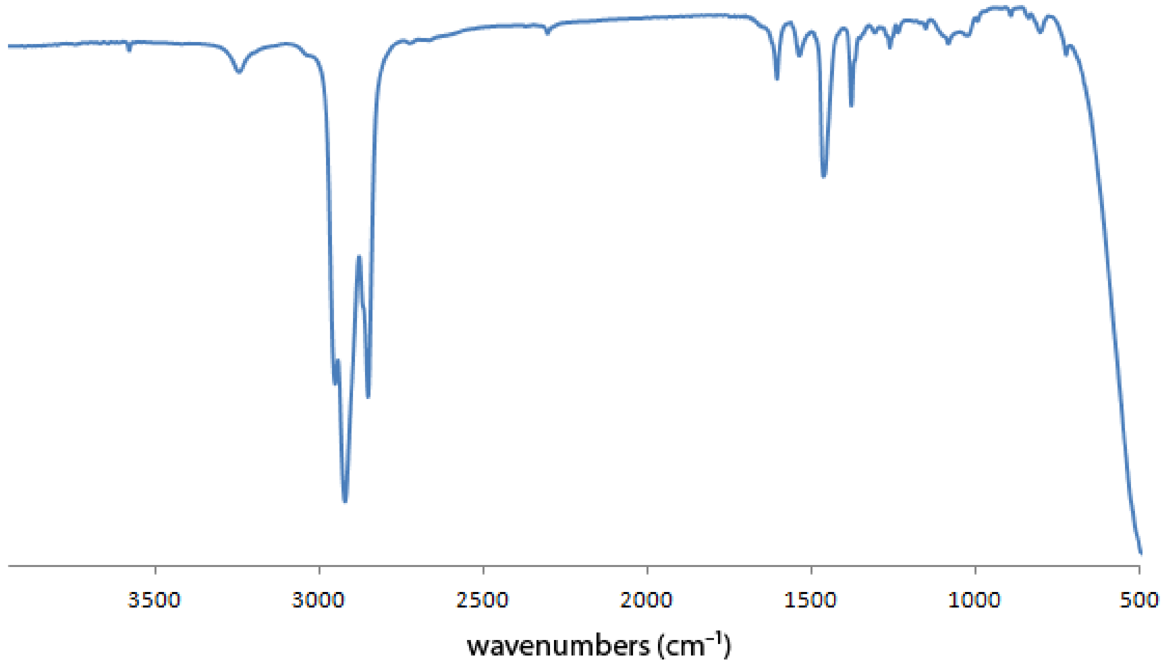

Figure S61. IR spectrum of **9** in a Nujol mull.

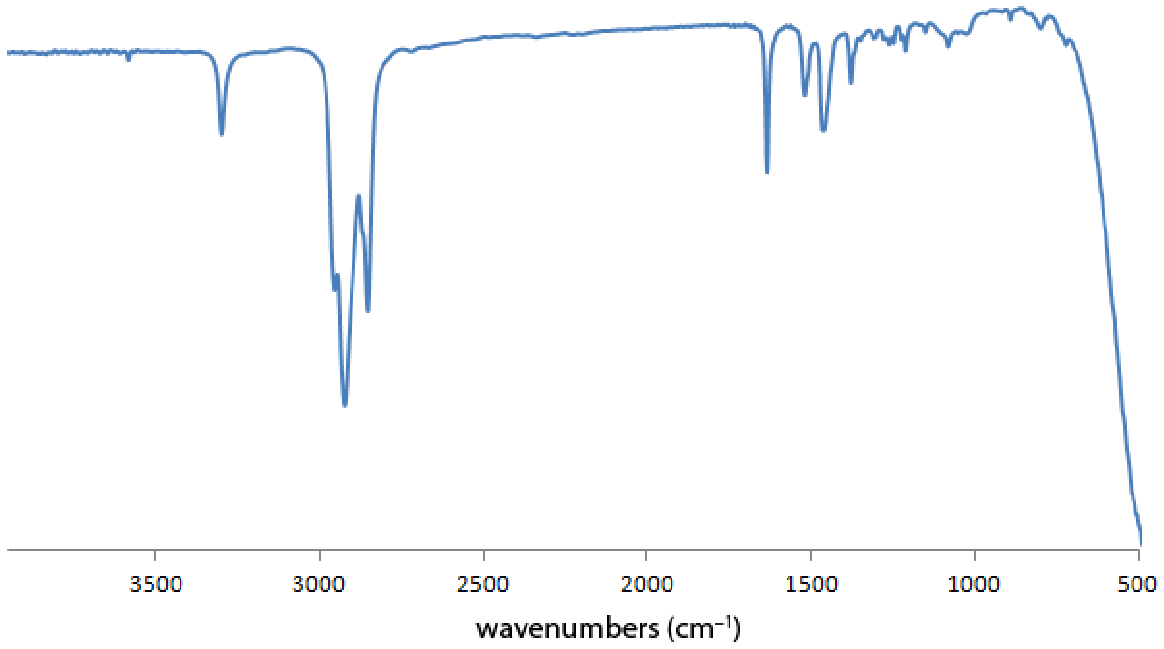

Figure S62. IR spectrum of **10** in a Nujol mull.

7. References

[1] A. R. Jupp, J. M. Goicoechea, *Angew. Chem. Int. Ed.* **2013**, *52*, 10064−10067.

[2] F. F. Puschmann, D. Stein, D. Heift, C. Hendriksen, Z. A. Gal, H.-F. Grützmacher, H. Grützmacher, *Angew. Chem. Int. Ed.* **2011**, *50*, 8420−8423.

[3] J. Cosier, A. M. Glazer, *J. Appl. Crystallogr.* **1986**, 105−107.

[4] *CrysAlisPro*, Agilent Technologies, Version 1.171.35.8.

[5] L. Palatinus, G. Chapuis, *J. Appl. Crystallogr.* **2007**, *40*, 786−790.

[6] a) G. M. Sheldrick, *Acta Crystallogr. Sect. A.* **1990**, *46*, 467−473; b) G. M. Sheldrick, *Acta Crystallogr. Sect. A.* **2008**, *64*, 112−122.

[7] a) G. te Velde, F. M. Bickelhaupt, E. J. Baerends, C. Fonseca Guerra, S. J. A. van Gisbergen, J. G. Snijders, T. Ziegler, *J. Comput. Chem.* **2001**, *22*, 931−967; b) C. Fonseca Guerra, J. G. Snijders, G. te Velde, E. J. Baerends, *Theor. Chem. Acc.* **1998**, *99*, 391−403; c) ADF2013.01, SCM, Theoretical Chemistry, Vrije Universiteit: Amsterdam, The Netherlands, http://www.scm.com.

[8] A. D. Becke, *J. Chem. Phys.* **1993**, *98*, 5648−5652.

[9] C. Lee, W. Yang, R. G. Parr, *Phys. Rev. B* **1988**, *37*, 785−789.

[10] S. H. Vosko, L. Wilk, M. Nusair, *Can. J. Phys.* **1980**, *58*, 1200−1211.

[11] a) E. van Lenthe, E. J. Baerends, J. G. Snijders, *J. Chem. Phys.* **1993**, *99*, 4597−4610; b) E. van Lenthe, E. J. Baerends, J. G. Snijders, *J. Chem. Phys.* **1994**, *101*, 9783−9792; c) E. van Lenthe, A. Ehlers, E. J. Baerends, *J. Chem. Phys.* **1999**, *110*, 8943−8953.

[12] A. Klamt, G. J. Schüürmann, *J. Chem. Soc., Perkin Trans. 2* **1993**, 799−805.

[13] L. Versluis, T. Ziegler, *J. Chem. Phys.* **1988**, *88*, 322−328.
